# Integration of transcriptomics, proteomics, and metabolomics data to reveal HER2-associated metabolic heterogeneity in gastric cancer with response to immunotherapy and neoadjuvant chemotherapy

**DOI:** 10.3389/fimmu.2022.951137

**Published:** 2022-08-04

**Authors:** Qihang Yuan, Dawei Deng, Chen Pan, Jie Ren, Tianfu Wei, Zeming Wu, Biao Zhang, Shuang Li, Peiyuan Yin, Dong Shang

**Affiliations:** ^1^ Department of General Surgery, First Affiliated Hospital of Dalian Medical University, Dalian, China; ^2^ Clinical Laboratory of Integrative Medicine, First Affiliated Hospital of Dalian Medical University, Dalian, China; ^3^ Department of Hepato-Biliary-Pancreas, Affiliated Hospital of North Sichuan Medical College, Nanchong, China; ^4^ Department of General Surgery, The First Affiliated Hospital of USTC, Division of Life Sciences and Medicine, University of Science and Technology of China, Hefei, China; ^5^ Department of Oncology, First Affiliated Hospital of Dalian Medical University, Dalian, China; ^6^ iPhenome Biotechnology (Yun Pu Kang) Inc., Dalian, China; ^7^ Institute of Integrative Medicine, Dalian Medical University, Dalian, China

**Keywords:** gastric cancer, HER2, multi-omics analysis, metabolic classification, precision medicine

## Abstract

**Background:**

Currently available prognostic tools and focused therapeutic methods result in unsatisfactory treatment of gastric cancer (GC). A deeper understanding of human epidermal growth factor receptor 2 (HER2)-coexpressed metabolic pathways may offer novel insights into tumour-intrinsic precision medicine.

**Methods:**

The integrated multi-omics strategies (including transcriptomics, proteomics and metabolomics) were applied to develop a novel metabolic classifier for gastric cancer. We integrated TCGA-STAD cohort (375 GC samples and 56753 genes) and TCPA-STAD cohort (392 GC samples and 218 proteins), and rated them as transcriptomics and proteomics data, resepectively. 224 matched blood samples of GC patients and healthy individuals were collected to carry out untargeted metabolomics analysis.

**Results:**

In this study, pan-cancer analysis highlighted the crucial role of ERBB2 in the immune microenvironment and metabolic remodelling. In addition, the metabolic landscape of GC indicated that alanine, aspartate and glutamate (AAG) metabolism was significantly associated with the prevalence and progression of GC. Weighted metabolite correlation network analysis revealed that glycolysis/gluconeogenesis (GG) and AAG metabolism served as HER2-coexpressed metabolic pathways. Consensus clustering was used to stratify patients with GC into four subtypes with different metabolic characteristics (i.e. quiescent, GG, AAG and mixed subtypes). The GG subtype was characterised by a lower level of ERBB2 expression, a higher proportion of the inflammatory phenotype and the worst prognosis. However, contradictory features were found in the mixed subtype with the best prognosis. The GG and mixed subtypes were found to be highly sensitive to chemotherapy, whereas the quiescent and AAG subtypes were more likely to benefit from immunotherapy.

**Conclusions:**

Transcriptomic and proteomic analyses highlighted the close association of HER-2 level with the immune status and metabolic features of patients with GC. Metabolomics analysis highlighted the co-expressed relationship between alanine, aspartate and glutamate and glycolysis/gluconeogenesis metabolisms and HER2 level in GC. The novel integrated multi-omics strategy used in this study may facilitate the development of a more tailored approach to GC therapy.

## 1. Introduction

Metabolic reprogramming is a characteristic shared by various solid tumours, which changes the availability of nutrients and the method of their utilisation by cells to meet the energy and material requirements of cancer (1–3). Oncogene-driven metabolic adaptations allow cancer cells to survive and thrive in the tumour microenvironment (4). Studies have shown that tumours have distinct substrate preferences for metabolites, which determines their metabolic heterogeneity (5, 6). The response of patients to chemotherapy and immunotherapy and their prognosis are intimately associated with tumour heterogeneity (7, 8). However, a method for distinguishing clinically relevant subtypes based on metabolic heterogeneity has not been established.

Gastric cancer (GC) is one of the most life-threatening malignancies, which is the third most common cause of cancer-related mortality worldwide (9). A majority of patients with GC have locally progressed or advanced disease at diagnosis. Systemic chemotherapy, radiotherapy, surgery, immunotherapy and targeted therapy are effective for the treatment of GC (10). However, GC is a highly diverse cancer with a complex genomic landscape of molecular changes, resulting in a wide range of treatment responses (11). Based on The Cancer Genome Atlas (TCGA) project, GC is divided into four subtypes, and promising therapeutic targets, such as human epidermal growth factor receptor 2 (HER2), have been identified (12, 13). HER2, commonly referred to as ERBB2, is a ligand-independent receptor tyrosine kinase, which is expressed in various epithelial cells and is involved in cell differentiation (14). Since its identification, HER2 has been reported to be amplified and overexpressed in various human malignancies, which is associated with a poorer prognosis, higher recurrence rates and shorter overall survival (15, 16). Trastuzumab, which targets HER2, is a first-line treatment for GC, and its combination with chemotherapeutic agents has significantly improved patient outcomes (17). In addition, trastuzumab is used to improve the efficacy of immunotherapy by inducing robust lymphocyte tumour infiltration (16). However, primary and acquired drug resistance remain major factors limiting its widespread use. Therefore, it is necessary to develop novel molecular subtypes based on HER2-associated molecular traits to facilitate clinical decision-making and improve precision medicine.

Previous studies have demonstrated that significant metabolic reprogramming occurs in patients with GC (18, 19). However, to the best of our knowledge, molecular typing of GC based on metabolic characteristics has not been reported. Metabolites are the ultimate functional products exhibiting genetic, protein and environmental changes (20). Metabolic subtypes facilitate clinical translation and provide novel insights into patient stratification for frontline therapies. In this study, we stratified GC into four metabolic subtypes with different metabolic traits based on novel multi-omics integration strategies and examined the pivotal role of the identified metabolic subtypes in the individualised treatment of GC.

## 2. Materials and methods

### 2.1. Study design and participants

A total of 112 patients with GC and 112 healthy volunteers were identified and enrolled from June 2021 to December 2021 at the First Affiliated Hospital of Dalian Medical University. This study was approved by the institutional ethics committee of The First Affiliated Hospital of Dalian Medical University (No. PJ-KS-KY-2021-93). All registered patients and healthy volunteers signed a written consent form authorising the use of their blood specimens for research purposes. The diagnosis of GC was confirmed in patients *via* gastroscopy or postoperative pathological analysis, and no indication of tumours was found in healthy individuals. All blood samples were collected preoperatively and after a 12-hour fast. Clinicopathological data were also collected, including age, sex, site of onset, TNM stage, metastatic location and histological differentiation. The following criteria were used to exclude participants from this study: (I) co-morbidity with other malignancies or a personal history of malignant tumours; (II) metabolic illnesses such as diabetes or hyperthyroidism; (III) recent persistent diarrhoea or vomiting and (IV) pregnant women, youngsters or substance abusers. **Table 1** summarises the clinical characteristics of the participants.

**Table:** Table 1. Clinical characteristics of the GC patients used for untargeted metabolomics.

	GC (n = 112)	Healthy individuals (n = 112)	p-value
**Gender (n,%)**			0.092
Male	78 (69.6%)	89 (79.5%)	
Female	34 (30.4%)	23 (20.5%)	
**Age (years, mean ± SD)**	63.32 ± 10.66	61.61 ± 11.79	0.255
**Hemoglobin (g/L, mean ± SD)**	123.01 ± 24.62	139.84 ± 35.19	<0.001
**Creatinine (μmol/L, mean ± SD)**	69.86 ± 19.45	72.62 ± 11.42	0.202
**Urea (mmol/L, mean ± SD)**	5.70 ± 1.64	5.47 ± 1.32	0.243
**Blood glucose (mmol/L, mean ± SD)**	5.41 ± 1.80	5.55 ± 1.34	0.526
**CEA**			
≤5	80 (71.4%)	–	
>5	23 (20.5%)	–	
NA	9 (8.0%)	–	
**CA19-9**			
≤27	84 (75.0%)	–	
>27	19 (17.0%)	–	
NA	9 (8.0%)	–	
**Grade**			
Low-grade intraepithelial neoplasia	2 (1.8%)	–	
High-grade intraepithelial neoplasia	3 (2.7%)	–	
Severe dysplasia	1 (0.9%)	–	
Early GC	9 (8%)	–	
Poorly differentiated	20 (17.9%)	–	
Moderately/poorly-differentiated	18 (16.1%)	–	
Moderately-differentiated	15 (13.4%)	–	
Well/moderately-differentiated	10 (8.9%)	–	
Well-differentiated	2 (1.8%)	–	
Signet ring cell carcinoma	6 (5.4%)	–	
NA	26 (23.2%)	–	
**Stage**			
Tis	1 (0.9%)	–	
I	44 (39.3%)	–	
II	10 (8.9%)	–	
III	31 (27.7%)	–	
IV	22 (19.6%)	–	
NA	4 (3.6%)	–	
**Type**			
Early GC	43 (38.4%)	–	
Adanced GC	69 (61.6%)	–	
**Immunohistochemistry**			
**GST-π**			
1	48 (42.9%)	–	
NA	64 (57.1%)	–	
**Her-2**			
0	30 (26.8%)	–	
1	11 (10.0%)	–	
2	5 (4.5%)	–	
3	5 (4.5%)	–	
NA	61 (54.5%)	–	
**Ki-67**			
≤25%	8 (7.1%)	–	
≤50%	10 (8.9%)	–	
≤75%	21 (18.8%)	–	
>75%	19 (17.0%)	–	
NA	54 (48.2%)	–	
**MHL-1**			
0	6 (5.4%)	–	
1	43 (38.4%)	–	
NA	63 (56.3%)	–	
**MSH-2**			
0	1 (0.9%)	–	
1	49 (43.8%)	–	
NA	62 (55.4%)	–	
**MSH-6**			
1	50 (44.6%)	–	
NA	62 (55.4%)	–	
**P53**			
Negative	10 (8.9%)	–	
Wild	27 (24.1%)	–	
Mutant	18 (16.1%)	–	
NA	57 (50.9%)	–	
**PMS-2**			
0	5 (4.5%)	–	
1	43 (38.4%)	–	
NA	64 (57.1%)	–	

NA. Not Available.

The immunohistochemical data of the 112 patients were also collected and curated postoperatively. The results were assessed by two experienced pathologists from the First Affiliated Hospital of Dalian Medical University who were blinded to the clinical outcomes. Because only some patients underwent immunohistochemical analysis postoperatively, we filtered the complete postoperative immunohistochemical information of only 51 patients with GC. The expression of HER2, P53, GST-π, Ki-67, MHL-1, MSH-2, MSH-6 and PMS-2 was evaluated based on the staining intensity and proportion of positive cells.

Transcriptomic (including >10,000 pan-cancer samples) and proteomic (including 392 GC samples) data were collected from TCGA, Genotype-Tissue Expression (GTEx) and The Cancer Proteome Atlas (TCPA) projects (21–23). The detailed processing of transcriptomic and proteomic data is shown in Supplementary Material A.

### 2.2. Processing of clinical samples and untargeted metabolomic strategies

To extract polar metabolites, 600 μL of methanol was mixed with 150 μL of serum sample to precipitate protein. The mixture was vortexed for 5 minutes and centrifuged at 5300 rpm for 20 minutes. Vacuum centrifugation was used to transfer and dry two replicates of 200 μL of supernatant. Polar metabolite samples were redissolved before their detection in positive and negative ion modes.

To extract lipids from serum, 120 μL of methanol was mixed with 20 μL of serum sample and vortexed for 3 minutes. The solution was then mixed with 360 μL of methyl tert-butyl ether and 100 μL of ultra-pure water. The solution was vortexed for 10 minutes and centrifuged at 13000 × g for 15 minutes. Similar to the abovementioned method of polar metabolite extraction, 200 μL of supernatant was transferred, dried and redissolved before lipidomic analysis.

Untargeted metabolomic analysis was performed on an UltiMate 3000 ultra-high-performance liquid chromatography system and a Q Exactive Quadrupole-Orbitrap High-Resolution Mass Spectrometer (Thermo Fisher Scientific, USA). The findings were used to annotate polar metabolites by searching the local database, mzCloud Library (Thermo Fisher Scientific, USA), Kyoto Encyclopedia of Genes and Genomes (KEGG) and Human Metabolic Group Database. In addition, the untargeted lipidomic data were analysed using the LipidSearch software (Thermo Fisher Scientific, USA). The accuracy of precursor mass was within 10 ppm for metabolite identification and structural annotation. The AUC values were extracted as the relative quantitative information of polar metabolites and lipids using the TraceFinder software (Thermo Fisher Scientific, USA).

## 3. Bioinformatic and statistical analyses

### 3.1 Pan-cancer analysis of ERBB2 highlighted its crucial role in the occurrence and progression of multiple human cancers

#### 3.1.1 ERBB2 expression analysis in pan-cancer

ERBB2 expression data were extracted from TCGA pan-cancer cohort and integrated using the Perl language. The wilcoxon test was performed to determine the differential expression of ERBB2 in various cancer types. A box plot was created using the R package ‘ggpubr’. A false discovery rate (FDR) of 0.05 was selected as the cut-off. The symbols ‘*’, ‘**’ and ‘***’ denote FDR values of <0.05, <0.01 and <0.001, respectively. Additionally, TCGA and GTEx datasets were also integrated to investigate and verify differences in ERBB2 expression between healthy and malignant tissues.

#### 3.1.2 Correlation of ERBB2 expression with clinical phenotype and prognosis

The association between clinical outcomes and ERBB2 expression was analysed across various cancer types using TCGA data. Disease-free interval (DFI), disease-specific survival (DSS), overall survival (OS) and progression-free interval (PFI) were used to evaluate the correlation between mRNA expression levels and survival rates using the ‘survival’ and ‘forestplot’ packages in R. Subsequently, the correlation between ERBB2 and clinicopathological parameters such as tumour grade, stage and status was analysed using the ‘limma’ and ‘ggpubr’ packages in R.

#### 3.1.3 Relationship between immunity and ERBB2 expression in pan-cancer

To elucidate the critical involvement of ERBB2 in the immunological milieu of different human malignancies, the association between ERBB2 expression and immunocyte infiltration was comprehensively analysed. Tumour IMmune Estimation Resource 2.0 (TIMER2.0; http://timer. cistrome.org/), based on the CIBERSORT, XCELL, EPIC, MCPCOUNTER and QUANTISEQ algorithms, was used to generate a detailed immune signature of tumour-infiltrating cells in various tumour samples from TCGA database. Subsequently, the association between ERBB2 expression and the abundance of tumour-infiltrating immune cells, such as CD4+ T cells, CD8+ T cells, B cells, neutrophils, dendritic cells (DCs) and macrophages, was analysed.

#### 3.1.4 Gene set enrichment analysis n pan-cancer

In view of the relationship between ERBB2 and tumour immunity and metabolism remains unclear, metabolism-related and immune-related pathways were identified using the ‘c2.cp.kegg.v7.4.symbols.gmt’ file from MsigDB (http://www.gsea-msigdb.org/). The activities of these pathways were evaluated using single-sample gene set enrichment analysis (ssGSEA) in R using the transcriptomic data of individual samples.

The samples of each tumour type were classified into two groups based on ERBB2 expression, with the top 30% and lowest 30% comprising the pathways related to ERBB2. GSEA was subsequently performed to examine the crucial role of ERBB2 in the immune and metabolic microenvironment of multiple human cancers.

### 3.2 Transcriptomic and proteomic analyses revealed the close association of ERBB2/HER2 with the immune microenvironment and metabolic remodelling of GC

To compute the immune score of each GC sample, the ‘GSVA’ package in R was used to perform ssGSEA using 29 immune gene sets reflecting diverse immune activities (24). Subsequently, Spearman correlation analysis was performed to examine the immunoregulatory role of ERBB2, GSTP1, MKI67, MLH1, MSH2, MSH6, PMS2 and TP53 in GC based on RNA-sequencing and protein chip data from TCGA and TCPA databases. The ssGSEA algorithm was used to compute the scores of immune- and metabolism-related pathways of each GC sample, which were further used to investigate the close association of ERBB2 with immune and metabolic pathways in GC.

### 3.3 Characterisation of metabolic landscape shifts during the tumourigenesis of GC

After the acquisition of metabolomic data, differentially expressed metabolites (DEMs) were determined between 224 matched blood samples collected from patients with GC and healthy individuals using the screening criteria of FDR < 0.05 and fold change > 1.2 or < 5/6. Subsequently, MetaboAnalyst5.0 (https://www.metaboanalyst.ca/) and MBROLE 2.0 (http://csbg.cnb.csic.es/mbrole2/) platforms were used to identify DEM-enriched metabolic pathways, which might play crucial roles in the tumourigenesis of GC (25, 26).

### 3.4 Weighted metabolite co-expression network analysis (WMCNA) identified hub metabolic pathways closely associated with HER-2 expression

Given the important role of ERBB2/HER2 in the immune microenvironment and metabolic remodelling of GC, HER2-coexpressed metabolites and their enriched metabolic pathways may be crucial for the pathophysiological processes of GC. The metabolomic and immunohistochemical (IHC) data of 51 patients with GC were integrated and subjected to WMCNA to determine HER2-coexpressed metabolites. The detailed process of WMCNA is described in Supplementary Material B.

To understand the possible significance of these HER2-coexpressed metabolites, their expression was examined in distinct HER2 subgroups and associated with clinical parameters (such as age, sex and tumour stage) and pathological markers (such as P53 and Kiki 67). Subsequently, the differential expression of these HER2-coexpressed metabolites was analysed in different tumour stages, grades and types. Metabolites related to clinical indicators were considered candidate metabolites. Furthermore, the differential expression of these candidate metabolites was analysed by integrating the metabonomic data derived from the blood samples of 112 patients with GC and 112 healthy volunteers. Differentially expressed metabolites closely associated with tumour stage, grade and type were eventually identified as the hub metabolites intimately related to the incidence and progression of GC.

Because the number of HER2-coexpressed metabolites was limited and the function of many metabolites remains unclear, pathway enrichment analysis performed using these metabolites alone may not yield accurate results. Therefore, we constructed a metabolite–metabolite interaction (MMI) network using Pearson correlation analysis and subsequently subjected it to pathway enrichment analysis using the MetaboAnalyst5.0 and MBROLE 2.0 platforms (25, 26).

### 3.5 Identification of the metabolic subgroups of GC based on the expression of alanine–aspartate–glutamate and glycolysis/gluconeogenesis metabolism-related genes

#### 3.5.1 Metabolic subgrouping and survival analysis

Genes belonging to the Molecular Signatures Database (MSigDB, http://www.gsea-msigdb.org/gsea/msigdb/search.jsp) gene sets ‘KEGG_ALANINE_ASPARTATE_AND_GLUTAMATE_METABOLISM’ and ‘KEGG_GLYCOLYSIS_GLUCONEOGENESIS’ were used as alanine–aspartate–glutamate (AAG) and glycolysis/gluconeogenesis (GG) metabolism-related genes, respectively. Consensus clustering was performed on AAG and GG metabolism-related genes using the ‘ConsensusClusterPlus’ package in R (parameters: reps = 100, pItem = 0.8, pFeature = 1). Ward.D2 and Pearson distances were used as the clustering algorithm and distance metrics, respectively (with k = 3). The median expression levels of coexpressed AAG and GG metabolism-related genes were used to divide samples into the quiescent (AAG ≤ 0, GG ≤ 0), AAG (AAG > 0, GG ≤ 0), GG (AAG ≤ 0, GG > 0) and mixed (AAG > 0, GG > 0) metabolic subgroups. Subsequently, Kaplan–Meier plots were generated to examine differences in survival (overall survival [OS] and disease-specific survival [DSS]) among different metabolic subgroups using the ‘survival’ and ‘survminer’ packages in R. Samples without follow-up information were excluded from survival analyses. In addition, ERBB2 expression was analysed across different metabolic subtypes.

#### 3.5.2 Integration of metabolic subtypes, immune subtypes and clinical traits

Consensus clustering was used to classify samples according to the immune subtypes identified by Thorsson et al. (27),who used immunogenomic techniques to assess the immunological tumour microenvironment and discovered six immune subgroups (wound healing [C1], IFN-γ dominant [C2], inflammatory phenotype [C3], lymphocyte depleted [C4], immunologically quiet [C5] and TGF-β dominant [C6]) using >10,000 tumour samples across 33 TCGA cancer types. The ‘circlize’, ‘gridBase’, ‘grid’, ‘ComplexHeatmap’ and ‘SimDesign’ packages in R were used to integrate metabolic subtypes, immune subtypes, tumour stage and tumour grade of GC samples, thus revealing the important association between tumour immunity and metabolism.

#### 3.5.3 Mechanism exploration

To examine the potential causes of prognostic differences among patients with different metabolic subtypes of GC, their mutation profiles, expression profiles (including mRNAs, miRNAs and lncRNAs) and immune microenvironment were intensively investigated. First, the metabolic typing data of each patient were integrated with the single nucleotide variant (SNV) and copy number variation (CNV) data retrieved from TCGA database, and TBtools was used to characterise the potential differences in mutation profiles among different metabolic subtypes (28). Subsequently, the expression profiles and clinical information of each patient, were filtered, and specific molecules among different metabolic subtypes were identified using the ‘limma’ package in R. Specific molecules were defined as follows: Molecules that were significantly upregulated in one subtype, when compared with the other three subtypes, were considered specific molecules of the subtype. The Cytoscape plug-ins ClueGO and CluePedia and yFiles Layout Algorithms were used to identify pathways enriched by these characteristic molecules to examine potential pathway disorders among different metabolic subtypes (29–31).

To examine differences in the immune microenvironment across different metabolic subtypes, various immune evaluation strategies were used. First, we assessed the proportion of immune components in the tumour microenvironment of different metabolic subtypes from a macroscopic perspective. The immunological score of each GC sample was calculated using the ‘estimate’ R package. Immune Cell Abundance Identifier (ImmuneCellAI, http://bioinfo.life.hust.edu.cn/ImmuCellAI#!/) is a technique that may be used to evaluate the level of immune cell infiltration (ICI) in diverse groups and predict the response of patients to immune checkpoint inhibitors (32). In addition to the ‘estimate’ algorithm, ImmuneCellAI was used to verify the abundance of ICI. The higher the immune score, the higher the level of ICI.

Furthermore, the infiltration abundance of each immune cell type was intensively investigated using various algorithms including TIMER, CIBERSORT, XCELL, EPIC, MCPCOUNTER and QUANTISEQ (33, 34). The ‘pheatmap’ R package was implemented to analyse the infiltration of diverse immune cells in each sample across different metabolic subtypes. Subsequently, the ‘kruskal.test’ function in R was used to compute statistical differences in ICI across different metabolic subtypes, and only immune cells with statistically significant changes (p < 0.05) were retained in the heatmap. In addition, the differential expression of common immune checkpoint genes was analysed across different metabolic subtypes, and only statistically significant results were visualised.

#### 3.5.4 Targeted drug sensitivity analysis and immunotherapy prediction

Given the prevalence of molecularly focused therapy for GC, the ‘pRRophetic’ package in R was used to estimate the medication sensitivity of each patient with GC based on their gene expression profiles (35). Subsequently, the ‘kruskal.test’ and ‘wilcox.test’ functions in R were used to screen potentially sensitive drugs among the four metabolic subtypes, with lower IC50 values indicating greater drug sensitivity.

The immunotherapeutic outcomes of each GC sample in different metabolic subtypes were predicted using the ImmuneCellAI algorithm. The ‘chisq.test’ function in R was used to investigate differences in immunotherapeutic responses among patients with GC with different metabolic characteristics. Subsequently, the Tumour Immune Dysfunction and Exclusion (TIDE, http://tide.dfci.harvard.edu/) and The Cancer Immunome Atlas (TCIA, https://tcia.at/home) platforms were used to evaluate immune escape potential and response to PD-1 and CTLA4 blockade therapy for each GC samples with different metabolic characteristics (36–38). The immunophenoscore (IPS) and exclusion scores of patients with GC were obtained from TCIA and TIDE platforms, respectively. The IPS of patients was determined by analysing the gene expression of four cell types that determine immunogenicity (immunosuppressive cells, MHC molecules, effector cells and immunomodulators). A higher IPS and a lower exclusion score predicted a better immunotherapeutic response.

### 3.6 Pan-cancer analysis reflected the overview of alanine–aspartate–glutamate and glycolysis/gluconeogenesis metabolism and their relationship with ERBB2 expression

In view of the crucial role of ERBB2 in pan-cancer, a systematic analysis of the potential role of metabolic pathways enriched by HER2-coexpressed metabolites in pan-cancer is also an important undertaking. To examine variations in AAG and GG metabolism-related genes in pan-cancer, the CNV and SNV data derived from TCGA database were analysed and visualised in heatmaps. Additionally, differential mRNA expression and methylation level were evaluated in pan-cancer. Furthermore, univariate Cox regression analysis was conducted to examine the prognostic significance of AAG and GG metabolism-related genes in various cancers. All of the abovementioned analyses were conducted using R and TBtools.

To examine the differential role of pathways influenced by AAG and GG metabolism in multiple human cancers, single-sample gene set enrichment analysis (ssGSEA) was used to compute the metabolic scores of each sample of each tumour. Based on the transcriptomic data of two tumour groups with the top and bottom 30% of metabolic scores, GSEA was used to examine differences in pathway activities.

Based on the metabonomic data, AAG and GG metabolic pathways were found to be closely associated with HER2 expression. In addition, the coexpression relationship between the ERBB2 gene and AAG and GG metabolism was validated at the transcriptomic level. The ‘cor.test’ function in R was used to analyse the correlation between AAG and GG metabolism pathway-related genes and the ERBB2 gene, and the ‘reshape2’ and ‘RcolorBrewer’ packages in R were used to visualise the results.

## 4. Results

### 4.1 Comprehensive characterisation of ERBB2 in pan-cancer highlights its pivotal role in the tumour microenvironment

**Figure 1:**
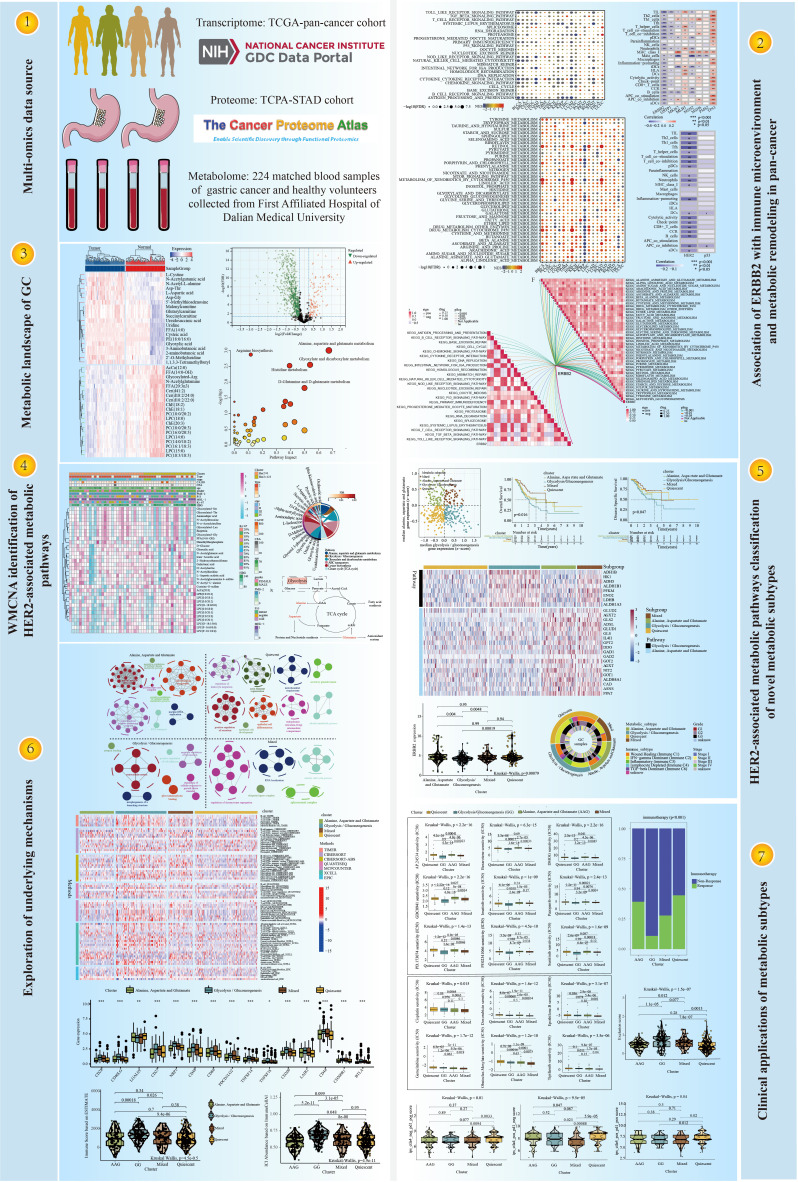
The workflow of this study.

The workflow of this study is displayed in **Figure 1**. A detailed description of the role of ERBB2 in multiple human cancers is shown in Supplementary Material C. We mainly emphasised the close association between ERBB2 expression and GC, especially for immune and metabolic traits. The mRNA level of ERBB2 was considerably elevated in most malignancies including GC (**Figure S1A**, **B**). In addition, a longer disease-free interval was substantially associated with increased ERBB2 expression (**Figure S1C**-**F**). Similarly, ERBB2 expression was lower in patients with G3 and G4 GC than in patients with G1 and G2 GC, which indicated that lower ERBB2 expression is associated with poor outcomes in patients with GC (**Figure S2**).

**Figure 2:**
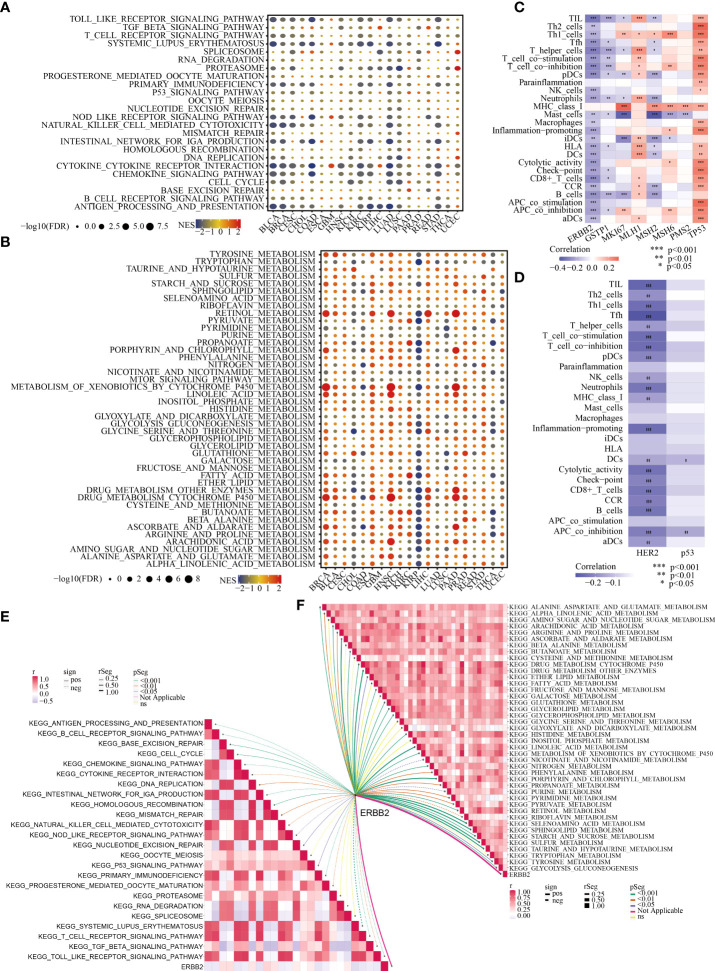
Association of ERBB2/HER2 with the immune microenvironment and metabolic remodelling in pan-cancer (especially in GC). Enrichment analysis for immune **(A)** and metabolic **(B)** pathways between tumour tissues with high and low ERBB2 expression; NES is the normalised enrichment score in the GSEA algorithm. ssGSEA highlights the regulatory role of ERBB2 **(C)** and HER2 **(D)** in the immune microenvironment of GC based on TCGA-STAD and TCPA-STAD cohorts. The correlation between ERBB2 and immune **(E)** and metabolic **(F)** pathways in GC was analysed.

Although there was no significant association between ERBB2 expression and the stage and recurrence of GC (**Figures S2**), ERBB2 was identified as a crucial regulator in the immune microenvironment and for the metabolic remodelling of GC (**Figures 2A**, **B**). As depicted in **Figure S3**, ERBB2 expression was negatively correlated with the infiltration levels of monocytes, M1 macrophages, M2 macrophages, myeloid dendritic cells, cancer-associated fibroblasts, naïve CD4+ T cells, CD8+ T cells, γδT cells, T helper 2 (Th2) cells and B cells but positively correlated with the infiltration levels of M0 macrophages, neutrophils, memory CD4+ T cells and T follicular helper (Tfh) cells in GC. In addition, ERBB2 expression was negatively correlated with many typical immune pathways in GC (e.g. antigen processing and presentation, cytokine receptor interaction, chemokine signalling pathway, natural killer (NK) cell-mediated cytotoxicity and Toll-like receptor signalling pathway) (**Figure 2A**). However, ERBB2 expression was positively associated with many typical metabolic pathways in GC (e.g. sulfur metabolism; sphingolipid metabolism; histidine metabolism; glycolysis/gluconeogenesis (GG) metabolism; glycerophospholipid metabolism and alanine, aspartate and glutamate (AAG) metabolism) (**Figure 2B**).

### 4.2 Transcriptomic and proteomic analyses reveal that ERBB2 and HER-2 are significantly associated with the immune status and metabolic features of patients with GC

In view of the crucial role of ERBB2 in the immune microenvironment and metabolic reprogramming of pan-cancer, the important relationship of the ERBB2 gene and its protein product HER2 with the immune and metabolic regulation of GC was extensively investigated. Based on TCGA-STAD and TCPA-STAD cohorts, we integrated the transcriptional and proteomic expression profiles of patients with GC and used the ssGSEA algorithm to calculate the immune cell infiltration (ICI) abundance and immune function score of each patient. Spearman correlation analysis revealed a substantial negative regulatory relationship between the ERBB2 gene and its protein product HER2 and the immunological state of patients with GC (**Figures 2C**, **D**). In addition, classical immune and metabolic pathways were identified using MsigDB. The complex relationship between ERBB2 and the regulation of these immune and metabolic pathways in GC was systematically analysed (**Figures 2E**, **F**). ERBB2 was found to be involved in the regulation of many immune pathways in GC, including antigen presentation, T- and B-cell receptor signalling pathways, cell cycle, chemokine signalling pathway, cytokine interaction, NK cell-mediated cytotoxicity, transforming growth factor beta signalling pathway and Toll-like receptor signalling pathway (**Figure 2E**). In addition, ERBB2 was found to play a pivotal role in the metabolic pathways of GC, including AAG, alpha linolenic acid, arginine and proline, glutathione, glycerolipid, histidine, pyrimidine and GG metabolism (**Figure 2F**).

### 4.3 Metabolic landscape shifts during the tumourigenesis of GC

**Figure 3:**
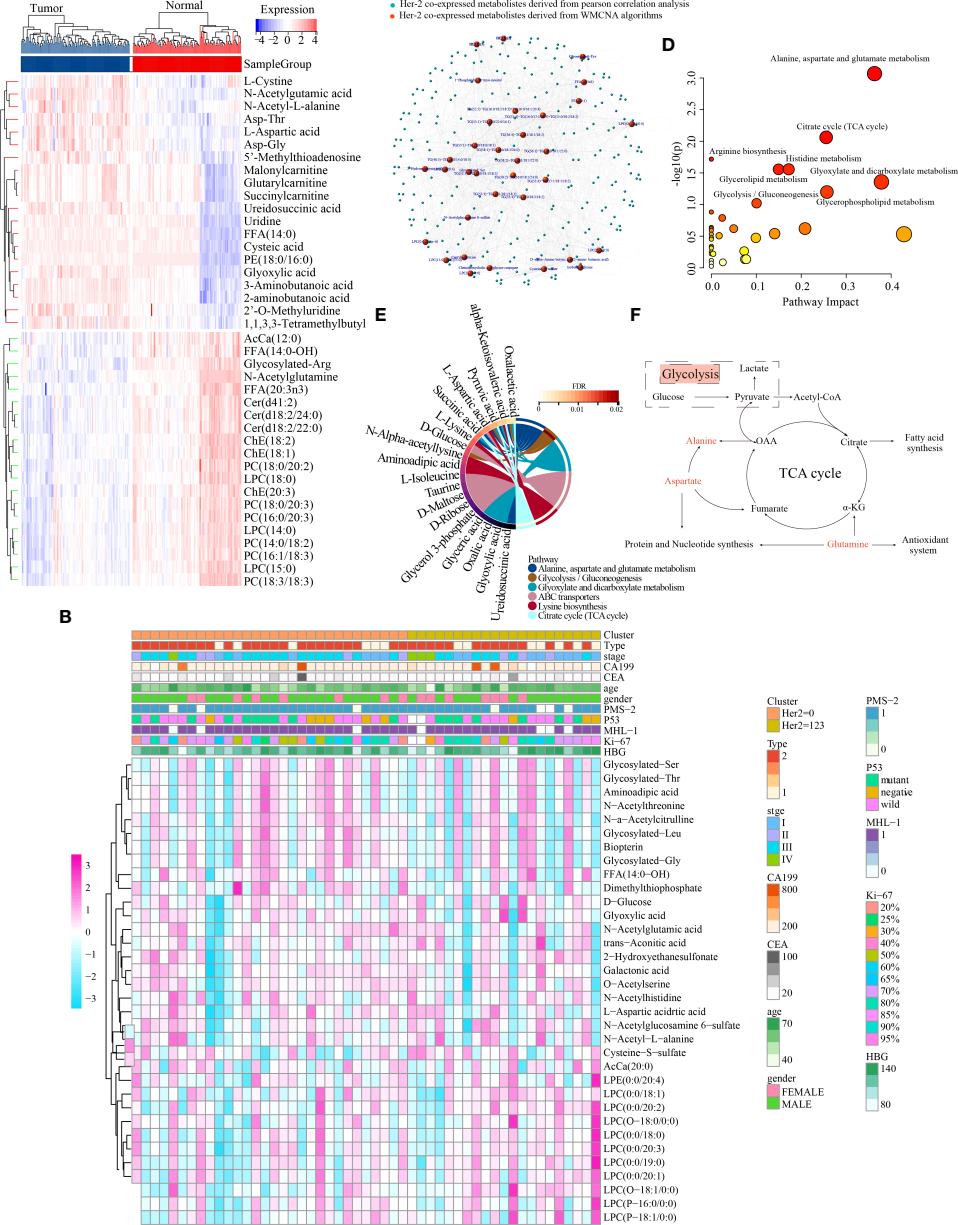
Characterisation of the metabolic landscape of GC and identification of HER2-coexpressed metabolites. **(A)** Differential expression heatmap of the top 40 metabolites. **(B)** The correlation of 34 HER2-coexpressed metabolites and clinicopathological characteristics in different clusters. **(C)** Development of a metabolite–metabolite interaction network. MetaboAnalyst5.0 **(D)** and MBROLE 2.0 **(E)** platforms were used to determine HER2-associated metabolic pathways. **(F)** Crosstalk between alanine–aspartate–glutamate and glycolysis/gluconeogenesis metabolism.

A human GC sample set was developed with sufficient fresh serum samples for complete metabolomic profiling of GC. This cohort included 112 patients with GC with different clinical stages and grades and 112 healthy volunteers. Mass spectrometry identified 1284 metabolites (1165 designated and 119 undesignated) using the blood samples. The ‘limma’ package in R identified 859 metabolites (129 upregulated and 730 downregulated) exhibiting distinct abundance between patients with GC and healthy individuals (FDR < 0.05, FC > 1.2 or <5/6) (**Figure S4A**, **Table S1**). In addition, numerous amino acids were highly represented and were abundant in tumours, including L-cystine, N-acetylglutamic acid, N-acetyl-L-alanine and L-aspartic acid (**Figure 3A**). These findings were consistent with those of a previous metabolomic study of 28 pairs of GC and healthy control samples (39). Moreover, many lipid metabolites were found to have a decreased level in GC (e.g. LPCs, PCs and Cers), which has been rarely reported previously.

To extensively analyse metabolic abnormalities associated with the pathogenesis of GC, KEGG pathway-based analysis was performed on DEMs among 224 matched peripheral blood samples. Both MetaboAnalyst 5.0 and MBROLE 2.0 platforms revealed that these DEMs were mainly enriched in AAG, arginine, glyoxylate and dicarboxylate metabolism (**Figure S4B** and **Table S2**).

### 4.4 Weighted metabolite co-expression network analysis identified hub metabolic pathways closely associated with HER2 expression

The average linkage strategy and Pearson correlation analysis were used to cluster 51 GC samples using the metabolomic data and IHC findings (**Figure S5A**). Pearson correlation analysis revealed no outliers. To ascertain whether the network was scale-free, we adopted the power of 4 (scale-free R^2^ = 0.88) as a soft-thresholding criterion (**Figure S5B**-**5D**). A total of 24 metabolite modules were preserved after average linkage hierarchical clustering (**Figures S5E**, **F**). The light cyan, midnight blue and black modules were significantly associated with HER2 expression, and 34 high-connection metabolites in these three modules were identified according to the requirements (i.e. MM > 0.7 and MES > 0.15) and preserved for further analysis (**Figure S5G**).

The distribution of the expression of these 34 metabolites and clinical traits of 51 GC samples across different HER2-expression subgroups is displayed in **Figure 3B**. Metabolites are substantially associated with clinical parameters such as tumour stage and grade and play an indispensable role in the pathophysiological process of disease. Therefore, we examined the potential relationship between these 34 metabolites and the stage, grade and type of tumour in 112 patients with GC and found that dimethylthiophosphate, glycosylated Leu, L-aspartic acid, LPC (0:0/19:0), N-acetylglucosamine-6-sulfate and N-acetyl-L-alanine were significantly associated with GC stage (**Figure S6A**). In addition, L-aspartic acid and N-acetylglucosamine-6-sulfate were significantly associated with GC grade (**Figure S6B**), whereas L-aspartic acid, N-acetylglucosamine-6-sulfate, O-acetylserine, and trans-aconitic acid were significantly associated with GC type (**Figure S6C**). Moreover, the serum levels of L-aspartic acid, N-acetyl-L-alanine and trans-aconitic acid were considerably higher, whereas those of glycosylated Leu and LPC (0:0/19:0) were dramatically lower in GC (**Figure S6D**).

Furthermore, Pearson correlation analysis was used to establish a MMI network (|Pearson’s correlation coefficient|>0.4) to highlight the important role of the abovementioned 34 HER2-coexpressed metabolites (**Figure 3C**). These metabolites were further submitted to the MetaboAnalyst 5.0 and MBROLE 2.0 platforms to identify the potential metabolic pathways closely associated with HER2 expression, and the results revealed that AAG and GG metabolism were strongly correlated with HER2 expression (**Figures 3D**, **E**, **Table S3**). The detailed association of GG metabolism with AAG metabolism is shown in **Figure 3F**.

### 4.5 Dual analysis of glycolysis/gluconeogenesis and alanine–aspartate–glutamate metabolism-related gene expression identified four distinct metabolic subgroups of GC

#### 4.5.1 Clustering and survival analyses

**Figure 4:**
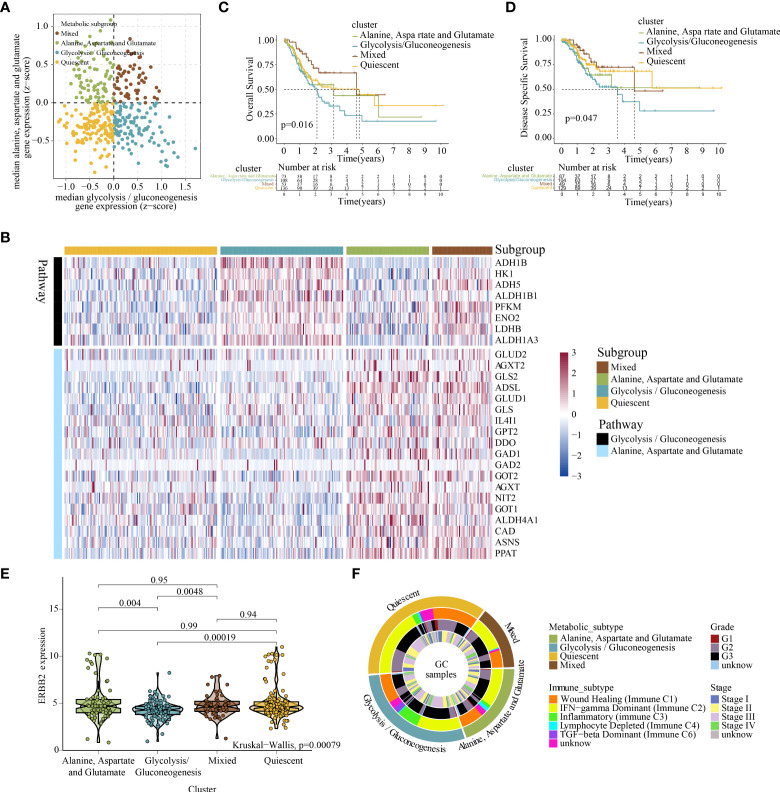
Classification of the metabolic subtypes of GC based on the expression of alanine–aspartate–glutamate and glycolysis/gluconeogenesis metabolism-related genes. **(A)** Scatter plot showing the median expression levels of coexpressed glycolysis/gluconeogenesis (X-axis) and alanine–aspartate–glutamate (Y-axis) metabolism-related genes in each GC sample. Metabolic subgroups were assigned based on the relative expression of glycolysis/gluconeogenesis and alanine–aspartate–glutamate metabolism-related genes. **(B)** Heatmap depicting the expression levels of coexpressed glycolysis/gluconeogenesis and alanine–aspartate–glutamate metabolism-related genes in each subgroup. **(C, D)** Kaplan–Meier survival analyses (OS and DSS) of patients with GC stratified based on metabolic subgroups. **(E)** Violin plot demonstrating ERBB2 expression in the four metabolic subtypes. **(F)** Overlay of metabolic subtypes (outer ring) with well-recognised immune subtypes of GC, tumour stage and tumour grade (inner rings).

RNA sequencing (RNA-seq) data from TCGA-STAD cohort were used to stratify GC depending on the expression profiles of GG and AAG metabolism-related genes. A previous study indicated that metabolic gene expression, including isoenzymes within particular pathways, differs among cancer types (40). Therefore, we used consensus clustering to recognise two groups of strongly coexpressed GG and AAG metabolism pathway-associated genes for metabolic subtyping and selecting genes coregulated within each pathway and related to GC biology. The median expression levels of coexpressed GG and AAG metabolism-related genes were computed for each sample and used to designate one of the following four metabolic profiles specifically relevant to these two pathways: quiescent, GG, AAG and mixed subtypes (**Figure 4A**). The expression profiles of GG and AAG metabolism-related genes across the metabolic subgroups are demonstrated in **Figure 4B**. Analysis of overall survival and disease-specific survival revealed that the prognosis of the four metabolic subgroups differed significantly (p = 0.016; p = 0.047), which indicated that the classification of the metabolic subtypes was clinically significant (**Figures 4C, D**). ERBB2 expression was lower in the GG metabolic subtype than in the other three subtypes (**Figure 4E**). To determine whether the expression patterns of the newly established metabolic subtypes could underlie differences among previously well-known immune subtypes, we investigated various GC immune subtypes for each sample in the study cohorts. As shown in **Figure 4F**, the circle diagram describes the distribution of tumour stage and grade across different metabolic subtypes. The GG subtype had a significant prevalence of the inflammatory phenotype, which may contribute to its poor prognosis.

#### 4.5.2 Mechanism exploration

**Figure 5:**
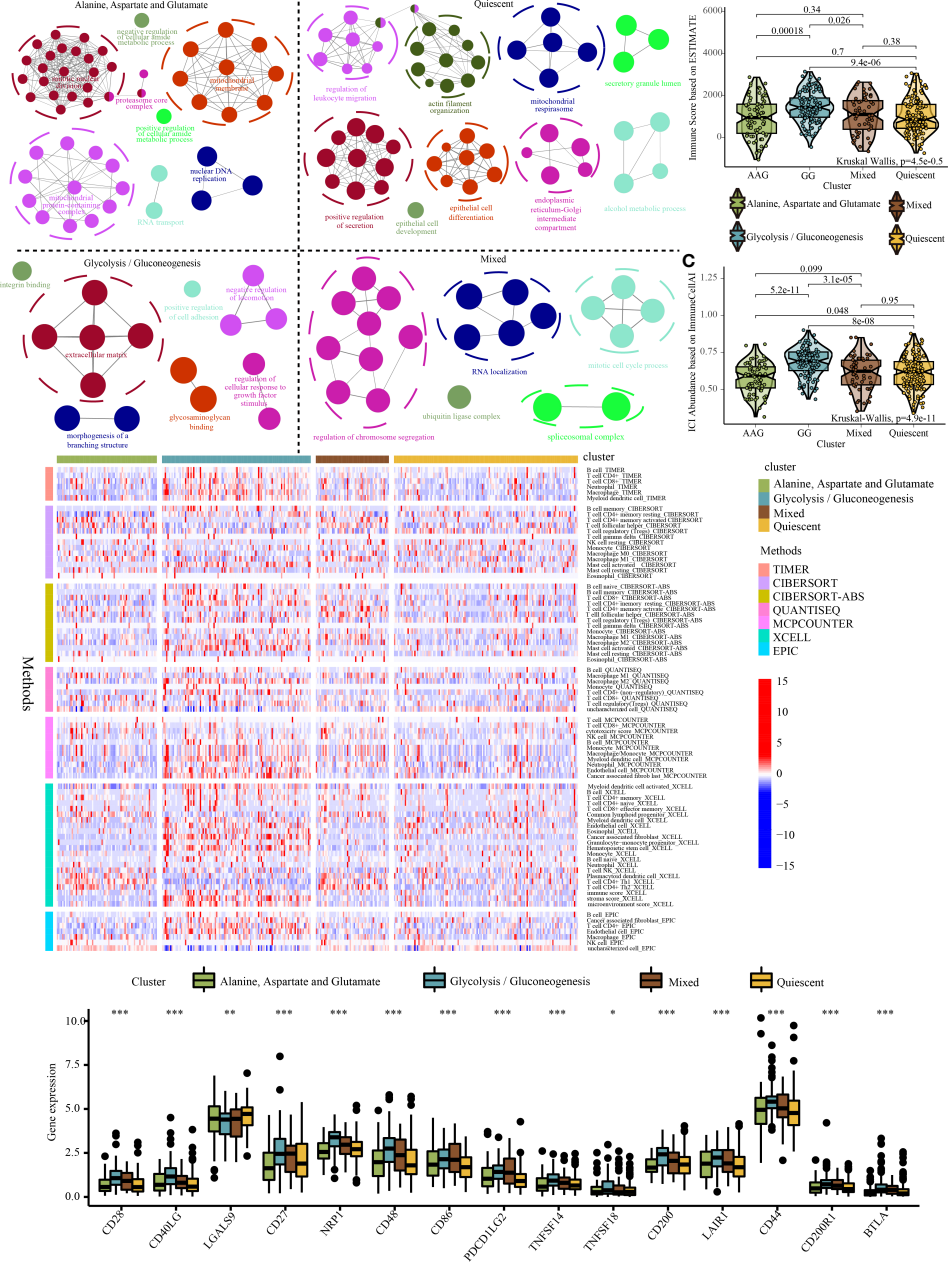
Systematic analysis of the specific molecule functions and tumour immune microenvironment across different metabolic subtypes. **(A)** Functional annotation of specific molecules of the alanine–aspartate–glutamate, glycolysis/gluconeogenesis, quiescent, and mixed subtypes. **(B)** Violin plot demonstrating immune scores of the four metabolic subtypes evaluated using the ESTIMATE algorithm. **(C)** Violin plot demonstrating the abundance of immune cell infiltration among the four metabolic subtypes evaluated using the ImmuneCellAI algorithm. **(D)** The distribution of immune cell infiltration among the four metabolic subtypes based on the TIMER, CIBERSOFT, QUANTISEQ, MCPCOUNTER, XCELL and EPIC algorithms (Note: Only immune cells with p < 0.05 were displayed in the heatmap). **(E)** Expression of immune checkpoint genes among the four metabolic subtypes. * indicates p <0.05; ** indicates p < 0.01; *** indicates p < 0.001.

Significant differences were found in survival outcomes among patients with different metabolic subtypes. Mutation profile analysis indicated some differences in SNV and CNV mutations among the four subtypes (**Figures S7A**-**C**). Although these differences were not significant, they may play a role in the prognosis of patients with different metabolic subtypes. In addition, we identified 673 specific molecules (involving 643 mRNAs, 16 miRNAs and 14 lncRNAs) for the quiescent subtype, 1627 specific molecules (involving 1478 mRNAs, 121 miRNAs and 28 lncRNAs) for the GG subtype, 1936 specific molecules (involving 1899 mRNAs, 1 miRNA and 36 lncRNAs) for the AAG subtype and 1655 specific molecules (involving 1627 mRNAs, 13 miRNAs and 15 lncRNAs) for the mixed subtype (**Figure S8**, Table S4–S7). Furthermore, the functional annotation of specific molecules of different metabolic subtypes was significantly different. The quiescent subtype was characterised by the regulation of leukocyte migration, epithelial cell development and differentiation, mitochondrial respirasome and endoplasmic reticulum–Golgi intermediate compartment (**Figure 5A**). The GG subtype was characterised by enhanced cell adhesion, integrin binding, glycosaminoglycan binding and regulation of cellular responses to growth factor stimuli (**Figure 5A**). The AAG subtype was mainly enriched in the regulation of cellular amide metabolism, mitochondrial protein-containing complex, nuclear DNA replication, mitotic nuclear division and proteasome core complex (**Figure 5A**). The mixed subtype was mainly enriched in the regulation of chromosome segregation, mitotic cell cycle process, spliceosomal complex and ubiquitin ligase complex (**Figure 5A**).

To further examine the abundance of immunocyte infiltration in the tumour microenvironment, various algorithms were used to estimate the levels of ICI across different metabolic subtypes. Both ESTIMATE and Immune Cell Abundance Identifier (ImmuneCellAI) algorithms revealed that a higher ICI score was detected in the GG subtype (**Figures 5B**, **C**). Similarly, other ICI evaluation algorithms (e.g. TIMER, CIBERSORT, QUANTISEQ, MCPCOUNTER, XCEL, and EPIC) indicated that the infiltration of cells involved in both innate and adaptive immune responses including macrophages, CD8+ T cells, CD4+ T cells and B cells was significantly higher in the GG subtype (**Figure 5D**). In addition, the GG subtype exhibited relatively higher expression levels of immune checkpoint genes (**Figure 5E**). The high infiltration of immune cells in the GG subtype may be a compensatory phenomenon in which the local immune function is suppressed by checkpoints, which may be responsible for the poor prognosis of the GG subtype.

### 4.6 Targeted drug sensitivity analysis and immunotherapy prediction

**Figure 6:**
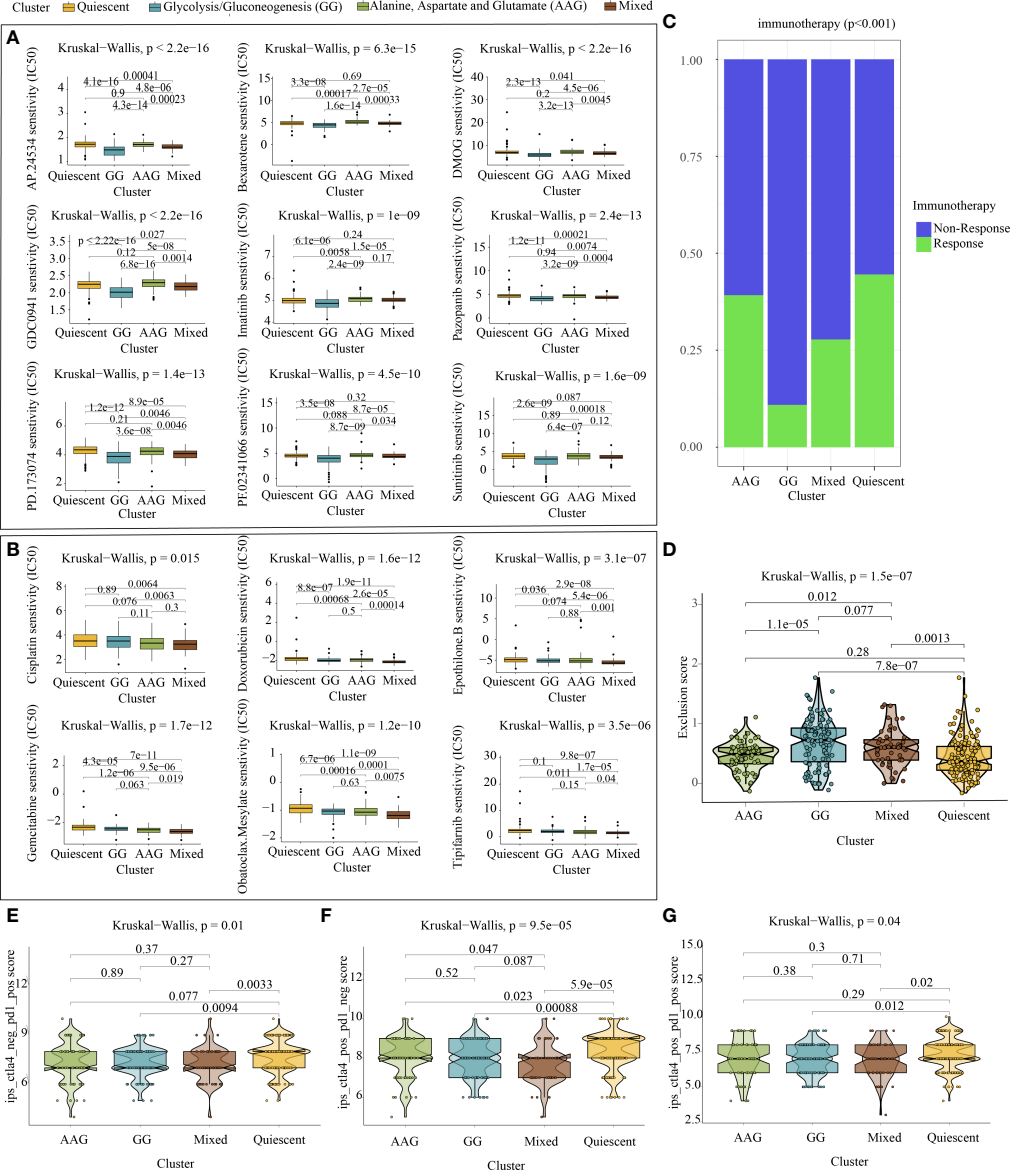
Chemotherapy prediction and immunotherapy response evaluation. **(A)** Identification of nine targeted drugs beneficial for the glycolysis/gluconeogenesis subtype. **(B)** Identification of six targeted drugs beneficial for the mixed subtype. **(C)** Prediction of immunotherapy outcomes of each metabolic subtype using the ImmuneCellAI algorithm. **(D)** Violin plot demonstrating the immune escape capacity of each metabolic subtype evaluated using the TIDE platform. **(E)** Distribution of IPS for PD1/PDL1/PDL2 inhibitors. **(F)** Distribution of IPS for CTLA4 inhibitors. **(G)** Distribution of IPS for CTLA4 and PD1/PDL1/PDL2 inhibitors.

Metabolic remodelling has profound implications for the prediction of chemotherapy response. The IC50 values of popular chemotherapeutics and targeted drugs were determined for each sample using the ‘pRRophetic’ R package. As shown in **Figure 6A**, the GG subtype had lower IC50 values for ponatinib (p < 2.2e−16), bexarotene (p = 6.3e−15), dimethyloxalylglycine (p < 2.2e−16), pictilisib (p < 2.2e−16), imatinib (p = 1e−09), pazopanib (p = 2.4e−13), PD173074 (p = 1.4e−13), crizotinib (p = 4.5e−10) and sunitinib (p = 1.6e−09), indicating that patients with this subtype may be extremely sensitive to these chemotherapeutic drugs. However, patients with the mixed subtype were more sensitive to cisplatin (p = 0.015), doxorubicin (p = 1.6e−12), epothilone B (p = 3.1e−07), gemcitabine (p = 1.7e−12), obatoclax mesylate (p = 1.2e−10) and tipifarnib (p = 3.5e−06) (**Figure 6B**).

As depicted in **Figure 6C**, analysis performed using ImmuneCellAI indicated that the quiescent and AAG subtypes had a more favourable immunotherapy response, suggesting that patients with GC with these subtypes might benefit from immune checkpoint blockade treatment. Similarly, analysis performed using TIDE demonstrated that the quiescent and AAG subtypes had a lower exclusion score, indicating that patients with GC with these subtypes were less likely to resist immunotherapy (**Figure 6D**). In addition, analysis performed using TCIA revealed that PD-1 blockage, CTLA4 blockage and joint blockage might be more beneficial for patients with the quiescent subtype (**Figures 6E**–**G**).

### 4.7 Pan-cancer characterisation of glycolysis/gluconeogenesis and alanine–aspartate–glutamate metabolism-related genes and their relationship with ERBB2 expression

Glucose and amino acid metabolism are important mechanisms of energy uptake by tumour cells. The results of previous analyses in this study validated that AAG metabolism is closely associated with the occurrence of GC, whereas AAG and GG metabolism are closely related to HER2 expression in GC. Moreover, patients with GC with different metabolic subtypes have different clinical outcomes and require different treatment strategies. Although many GG and AAG metabolism-related genes have been explored in tumours, the pan-cancer characterisation of GG and AAG metabolism-related genes is not well summarised. Therefore, we examined the genomic and transcriptomic data, including mRNA expression, SNV, CNV, prognostic values and DNA methylation levels of tumour and healthy tissues across 20 cancer types (**Figure S9**, **S10**). GSEA was used to investigate the related cell signalling of AAG and GG metabolism in each cancer type based on the transcriptome of two tumour groups with the top and bottom 30% of metabolism scores. AAG and GG metabolism were found to be closely associated with the hallmarks of classical oncogenic and metabolic pathways (**Figure S9**, **S10**). Previous metabonomic data revealed a close relationship between GG and AAG metabolism and HER2 expression. In addition, pan-cancer transcriptomic data validated the close relationship between GG and AAG metabolism-related genes and ERBB2 expression (**Figure S11**).

## 5. Discussion

GC is distinguished by tumour heterogeneity at the genetic, histological and phenotypic levels (41). Precise molecular characterisation and customised therapies are critical for the treatment of GC. Biomarkers, particularly HER2, are increasingly used to guide systemic therapeutic methods and help in the identification of patients with GC who may respond to immunotherapy and targeted therapy (42). Although the Lauren and the World Health Organisation classification are two of the most widely used classification systems for GC, they remain inadequate for individualised therapy (43). Recent advancements in multi-omics technologies have facilitated the investigation of GC at high resolution and at the molecular level. Multi-omics data integration strategies across multiple cellular function levels provide unprecedented insights into the underlying pathophysiological mechanisms of cancers and facilitate tumour classification, diagnosis and prognosis (44–47). In the present study, based on HER2-related metabolic pathways, we developed a novel multi-omics integration strategy to reveal the metabolic heterogeneity of GC and its clinical application value.

HER2 serves as a routine immunohistochemical indicator for GC postoperatively and is critical in the assessment of prognosis and targeted pharmacological intervention. In a *post-hoc* study of patients with HER2 immunohistochemical scores of 3+ or 2+ and fluorescence *in situ* hybridisation-positive tumours, trastuzumab in combination with chemotherapy improved the median overall survival compared with chemotherapy alone (16.0 versus 11.8 months, respectively) (48). Given the crucial role of HER2 in individualised tumour intervention, we first characterised ERBB2 in multiple human cancers in a multifaceted manner. The findings revealed that ERBB2 expression was significantly upregulated in most tumour types and was closely associated with tumour stage, grade, recurrence, prognosis, immune microenvironment and metabolic reprogramming. In addition, increased ERBB2 expression was closely associated with a longer disease-free period in patients with GC. More importantly, ERBB2 expression negatively regulated various innate and adaptive immune responses but was positively correlated with the activation of GG, AAG, sulfur and glycerophospholipid metabolism in GC.

To examine the overall metabolic landscape of GC, we performed metabolic profiling of 112 patients with GC and 112 healthy individuals using their serum samples. The results revealed metabolic shifts during the pathogenesis of GC and identified a network of metabolic shifts associated with the emergence and development of GC, including AAG, arginine, glyoxylate and dicarboxylate metabolism. WMCNA was used to determine three metabolic modules involving 34 candidate metabolites that were significantly associated with HER2 expression. Among these coexpressed HER2 metabolites, L-aspartic acid, a component of the AAG metabolic pathway, was suggested to be the central metabolite in the pathophysiological process of GC. Significant upregulation of L−aspartic acid in GC was found to be closely associated with the stage, grade and type of tumour and HER2 expression. In addition, these HER2-coexpressed metabolites were predominantly enriched in AAG and GG metabolism, which was consistent with the results of the transcriptomic analysis.

Tumours are both proliferative and metabolic. The uncontrolled development of a tumour necessitates the requirement of a vast amount of nutritious components (49). In this study, HER2-related metabolic subtypes were clustered based on multi-omics data and were found to be mainly related to energy metabolism and anabolism. GC has distinct metabolic profiles based on the expression of genes involved in GG and AAG metabolism, which affects clinical outcomes and supports the concept of targeting tumour metabolic plasticity as a method of reprogramming an aggressive tumour type.

The Warburg effect refers to the increase in the rate of glycolysis and anabolism, which is a typical metabolic feature of tumours. In most tumours, high glycolysis is accompanied by upregulation of oxidative phosphorylation, ensuring the production of energy required for rapid tumour proliferation (50, 51). At present, inhibition of oxidative phosphorylation is a promising strategy for targeting cancer. Excess glycolytic intermediates are converted to amino acids, nucleotides and lipids, which are necessary to support cell growth, accompanied by high lactic acid accumulation. Metastasis is the main cause of the failure of cancer treatment. High concentrations of lactic acid in the tumour microenvironment can contribute to tumour metastasis. Malignant melanomas with high expression of MCT1 can resist oxidative stress through excessive intake of lactic acid, thus gaining invasion and metastasis abilities (52). In addition, lactic acid generated during glycolysis in cancer cells phosphorylates the transcription factor TFEB by activating mToR1 and inhibits the degradation of HIF-2α lysosomes, thus promoting the reprogramming of tumour-associated macrophages and optimising the microenvironment for tumour growth (53). Moreover, lactate acts as an intrinsic inflammatory mediator, promoting the synthesis of interleukin (IL)-17A by T cells and macrophages, thus promoting chronic inflammation in the tumour microenvironment (54). The interaction between tumour metabolites and immune cells shows that lactate may contribute to immunological escape (55). Extracellular lactate can block the development of monocytes to dendritic cells (DCs) and inactivate the production of cytokines by DCs and cytotoxic T cells, which are critical for the antitumoural response (56, 57). Similarly, the findings of this study revealed that GC with a glycolytic phenotype had higher inflammation and a lower ERBB2 expression level, which was consistent with the role of glycolysis in tumour aggressiveness and the inflammatory milieu in GC.

Many non-essential amino acids are essential for cell growth and serve as major carbon sources for tumour metabolism. There are significant differences in glutamine uptake between the tumour microenvironment and normal tissues. Studies have shown that glutamine metabolism in the tumour microenvironment promotes tumour growth but impairs the antitumour activity of immune cells (58). Glutamine not only provides ATP for cells but also acts as an important carbon source for the synthesis of lipids and non-essential amino acids. Glutamine-derived carbon is the major substrate for *de novo* lipid synthesis, whereas excess carbon and ammonia are safely removed from cells to avoid the accumulation of ammonia (58). Glucose and glutamine are both prominent substrates for the intracellular hexosamine biosynthetic pathway (HBP), which is a key pathway for the generation of glycosyl-based donors. SRPK2 is glycosylated to regulate *de novo* lipid synthesis in tumours (59). The prognosis of GC subtypes dependent on AAG metabolism is also poor. In this study, the mixed subtype had the best outcome of all four subtypes. The possible reasons for this phenomenon are as follows: Glutamine and aspartic acid can be metabolised to alanine, which inhibits pyruvate kinase activity and glycolysis in many different cells (60). In this study, the glucose and amino acid metabolism of patients with GC with the mixed subtype appeared to be exuberant. These two metabolic pathways antagonise each other and maintain the metabolic balance within the tumour. Therefore, patients with the mixed subtype had better prognostic characteristics.

Furthermore, we examined the potential causes of prognostic differences among different metabolic subtypes at the transcriptomic level. First, we examined CNVs and SNVs in the four metabolic subtypes and found slight differences in the mutational spectrum of patients with GC with different metabolic characteristics. Subsequently, functional enrichment analysis of the specific molecules of each metabolic subtype revealed that the GG subtype was closely related to cell adhesion, integrin binding, glycosaminoglycan binding and growth factor response, suggesting its association with a poor prognosis. In addition, the findings revealed that the tumour microenvironment of different metabolic subtypes showed evident heterogeneity, and the GG subtype showed higher levels of immune cell infiltration and expression of immune checkpoint genes. One of the reasons for the worst prognosis of patients with the GG subtype is that the overactivity of immune checkpoints weakens the anti-tumour immune response in the tumour microenvironment. In addition, the high infiltration of immune cells is a compensatory phenomenon of local immune incompetence.

Based on targeted drug analysis and prediction of immunotherapy response in patients with GC with different metabolic subtypes, we found that chemotherapy or targeted therapy was more likely to be beneficial for the mixed and GG subtypes, whereas immunotherapy might improve clinical outcomes in patients with the AAG and quiescent subtypes. Specifically, the glycolysis subtype was more sensitive to ponatinib, bexarotene, dimethyloxalylglycine, pictilisib, imatinib, pazopanib, PD173074, crizotinib and sunitinib; however, the mixed subtype was more sensitive to cisplatin, epothilone B, gemcitabine, obatoclax mesylate and tipifarnib. The quiescent subtype might benefit more from PD-1 and CTLA4 inhibitors.

Finally, we examined genes regulating glycolysis and alanine–aspartic acid–glutamate metabolism. The identified metabolic regulatory genes had evident mutation characteristics in pan-cancer, especially GC; had differential expression in many tumour types and were closely associated with the clinical outcomes of tumours. In addition, the close relationship between GG and AAG metabolism-related genes and ERBB2 expression was validated at the pan-cancer transcriptomic level. These findings may provide valuable data for further study of glucose and amino acid metabolism in other tumour types.

## 6. Conclusions

Transcriptomic and proteomic analyses highlight the close association of HER2 level with the immune status and metabolic features of patients with GC. Metabolomics analysis highlights the co-expressed relationship between alanine, aspartate and glutamate and glycolysis/gluconeogenesis metabolisms and HER2 level in GC. The novel multi-omics integration strategy used in this study successfully identified four types of GC populations with different metabolic characteristics based on HER2-associated metabolic pathways. The GG subtype was characterised by lower ERBB2 expression, higher inflammation and a poor prognosis. Contradictory features were determined for the mixed subtype with the best prognosis. The GG and mixed subtypes were highly sensitive to chemotherapy, whereas the quiescent and AAG subtypes were more likely to benefit from immune checkpoint inhibitors.

## Data availability statement

The original contributions presented in the study are included in the article/**Supplementary Material**. Further inquiries can be directed to the corresponding authors.

## Ethics statement

All registered GC patients and healthy volunteers signed a consent form authorizing the use of their blood specimens for research purposes. This study was approved by the institutional ethics committee of The First Affiliated Hospital of Dalian Medical University (No. PJ-KS-KY-2021-93). The patients/participants provided their written informed consent to participate in this study.

## Author contributions

The content and authorship of the paper are entirely the responsibility of the writers themselves. QY and DD contributed to the design of the study, collection, and interpretation of data, and drafting and revising the manuscript. CP, JR, TW, ZW, BZ, and SL participated in the collection and analysis of data. PY and DS are responsible for the design of the study and reviewed/edited the manuscript. All authors contributed to the article and approved the submitted version.

## Funding

This research was funded by The Key Research and Development Project of Liaoning Province (No. 2018225054).

## Acknowledgments

The authors expressed gratitude to clinical collaborators and data producers at TCGA, GTEx, and TCPA platforms. We also thank Bullet Edits Limited for the linguistic editing of the manuscript.

## Conflict of interest

ZW and PY are co-founders of iphenome (Yun Pu Kang) biotechnology Inc. ZW is an employee of iphenome (Yun Pu Kang) biotechnology Inc.

The remaining authors declare that the research was conducted in the absence of any commercial or financial relationships that could be construed as a potential conflict of interest.

## Publisher’s note

All claims expressed in this article are solely those of the authors and do not necessarily represent those of their affiliated organizations, or those of the publisher, the editors and the reviewers. Any product that may be evaluated in this article, or claim that may be made by its manufacturer, is not guaranteed or endorsed by the publisher.

## Supplementary material

Supplementary Figure 1 The mRNA expression profiles and prognostic performance of ERBB2 in pan-cancer. **(A)** The mRNA expression of ERBB2 between tumour and healthy tissues was assessed using tissues from TCGA database. **(B)** Comparison of ERBB2 expression levels between tumour tissues from TCGA database and healthy tissues from the GTEx database. Relationship between ERBB2 expression and overall survival **(C)**, disease-free interval **(D)**, disease-specific survival **(E)** and progression-free interval **(F)** in pan-cancer.

Supplementary Figure 2 Correlation between ERBB2 expression and tumour stage, tumour grade and tumour status.

Supplementary Figure 3 Correlation between ERBB2 expression and immune infiltration in pan-cancer.

Supplementary Figure 4 Identification of differentially expressed metabolites and their related metabolic pathways. **(A)** Volcano plot demonstrating the expression profile of 1165 metabolites; a total of 859 metabolites exhibited significant differential abundance (FDR < 0.05, fold change > 1.2 or < 5/6) in the peripheral blood samples of patients with GC and healthy volunteers. **(B)** Topology analysis of dysregulated metabolic pathways associated with the occurrence of GC. The size of the bubble area denotes the impact of each pathway, with a colour representing significance from the highest in red to the lowest in white.

Supplementary Figure 5 Detailed processes of weighted metabolite co-expression network analysis. **(A)** Clustering dendrogram of 51 samples to detect outliers (white-to-red linear gradient colour associated with corresponding HER2 expression). **(B)** The scale-free fit index for soft-thresholding powers (β). **(C)** Mean connectivity calculation. **(D)** Evaluation of scale-free topology with a β of 4. **(E)** Hierarchical clustering dendrograms of the identified coexpressed metabolites in specific modules of GC. **(F)** Distribution of average metabolite significance and errors in modules associated with HER2 expression. **(G)** Heatmap demonstrating the correlation between module eigenmetabolites and HER2 expression.

Supplementary Figure 6 Identification of differentially expressed hub metabolites significantly associated with GC stage, grade and type. Correlation between 34 HER2-coexpressed metabolites and tumour **(A)** stage, **(B)** grade and **(C)** type. **(D)** The expression distribution of the HER2-coexpressed metabolites associated with tumour stage, grade and type in tumour and healthy samples. Only metabolites with a p-value of <0.05 are demonstrated.

Supplementary Figure 7 Panoramic view of the **(A)** SNV, **(B)** CNV amplification and **(C)** CNV deletion frequencies of the top 20 mutated genes across the four metabolic subtypes.

Supplementary Figure 8 Panoramic view of the specific molecules **(A**: mRNA, **B**: miRNA, **C**: lncRNA**)** of each metabolic subtype.

Supplementary Figure 9 Panoramic view of glycolysis/gluconeogenesis metabolism-related genes in pan-cancer. **(A)** Changes in the mRNA expression of glycolysis/gluconeogenesis metabolism-related genes across cancer types. The frequencies of copy number variation **(B)** and single-nucleotide variation **(C)** in diverse types of cancers. **(D)** Enrichment analysis for cancer pathway signalling between tumour samples with high and low enrichment scores of the genes. **(E)** Survival landscape of the genes across cancer types. **(F)** Heatmap demonstrating the methylation levels of the genes across cancer types.

Supplementary Figure 10 Panoramic view of alanine–aspartate–glutamate metabolism-related genes in pan-cancer. **(A)** Changes in the mRNA expression of alanine–aspartate–glutamate metabolism-related genes across cancer types. The frequencies of copy number variation **(B)** and single-nucleotide variation **(C)** in diverse types of cancers. **(D)** Enrichment analysis for cancer pathway signalling between tumour samples with high and low scores of the genes. **(E)** Survival landscape of the genes across cancer types. **(F)** Heatmap demonstrating the methylation levels of the genes across cancer types.

Supplementary Figure 11 Relationship between ERBB2 expression and glycolysis/gluconeogenesis **(A)** and alanine–aspartate–glutamate **(B)** metabolism.
