# Supplementary material for: Integration of transcriptomics, proteomics, and metabolomics data to reveal HER2-associated metabolic heterogeneity in gastric cancer with response to immunotherapy and neoadjuvant chemotherapy
Source: Front Immunol. 2022 Aug 4;13:951137. doi: 10.3389/fimmu.2022.951137 (PMC9389544; doi:10.3389/fimmu.2022.951137)
Supplement: Supplementary file 1 [file DataSheet_1.docx]

**Contents of Supplementary Materials**

**Supplementary Material A: Detailed processing of transcriptomic and proteomic data --------------------------------------------------------------------------------------------2**

**Supplementary Material B: Detailed processing of weighted metabolite correlation network analysis -------------------------------------------------------------3**

**Supplementary Material C: Detailed description of the role of ERBB2 in multiple human cancers -----------------------------------------------------------------------------4–6**

**Supplementary Material A**

**Detailed processing of transcriptomic and proteomic data**

The expression data of related cancers and adjoining normal samples along with information outlining related clinicopathological features were extracted from The Cancer Genome Atlas (TCGA) database, which is a landmark cancer genomic project comprising a large number of cancer samples spanning 33 cancer types. The Genotype-Tissue Expression (GTEx) project, which is currently working to provide a comprehensive public resource for studying tissue-specific gene expression and regulation, was used for enrichment analysis of tissue-specific normal samples and those obtained from TCGA. The pan-cancer transcriptomic RNA-seq and clinical data were obtained from TCGA and GTEx databases using the UCSC Xena platform (<https://xenabrowser.net/>).

The Cancer Proteome Atlas (TCPA), a user-friendly data portal, was created in 2013 to make cancer proteomic datasets more accessible to the larger scientific community by combining RPPA chip data from TCGA with data from numerous oncology research initiatives. TCPA delivers level 3 and 4 data for each dataset. Level 3 data include normalised data from independent batches, whereas level 4 data represent aggregated data from numerous batches. Level 4 gastric cancer data containing 392 tumour samples and 218 protein expression profiles were used as the proteomic data and preserved for further analysis. The RNA-sequencing data and the corresponding clinical information of TCGA-STAD cohort were used as the transcriptomic data.

**Supplementary Material B**

**The detailed process of weighted metabolite correlation network analysis**

The ‘GoodSamplesGenes’ function from the ‘WGCNA’ package was used to verify the quality of raw data. Subsequently, Pearson correlation analysis was used to create an adjacency matrix. A soft-thresholding parameter was then used to ensure a scale-free coexpression network. Additionally, hierarchical clustering of the weighting coefficient matrix was used in WMCNA to identify metabolite modules, which were clusters of highly related metabolites. To analyse the association among functional modules, the adjacency matrix was converted to a topological overlap measure (TOM) matrix. A clustering dendrogram of the TOM matrix was constructed through average linkage hierarchical clustering. To produce acceptable modules, the minimum metabolite module size was set to 20, and the ‘DynamicTreeCut’ approach was used to categorise metabolites with similar expression patterns into the same metabolite modules.

Module eigenmetabolites (MEMs) and metabolite significance (MES) were used to define modules associated with HER2 expression. MEMs were considered the primary component of each module, and MEM expression was acknowledged on behalf of all metabolites included within a particular module. Therefore, the correlation between MEMs and HER2 was analysed to determine the clinically significant module. Additionally, MES was defined as the mediating p-value for each metabolite in the linear regression analysis of clinical features and metabolite expression patterns. The term ‘module significance’ (MS) refers to the average MES of modules across all metabolites. Clinically relevant modules (CRMs) were defined as those with MS of <0.05. To determine the relevance of each metabolite in these modules, metabolite module membership (MM) was evaluated in each CRM. Metabolites with |MES| of >0.15 and |MM| of >0.7 were considered HER2-coexpressed metabolites.

**Supplementary Material C**

**Detailed description of the role of ERBB2 in multiple human cancers**

Pan-cancer cohorts derived from TCGA database revealed that the mRNA expression of ERBB2 was considerably higher in most malignancies such as BLCA, BRCA, CESC, CHOL, GBM, LIHC, LUAD, STAD, THCA and UCEC than in matched adjacent healthy tissues (Supplementary Figure 1A). However, ERBB2 expression was low in various cancers, including COAD, HNSC, KICH, KIRC and KIRP (Supplementary Figure 1A). Owing to the minimal number of healthy tissue samples in TCGA database, TCGA and GTEx data were merged to examine the differential expression of ERBB2 in various healthy and cancer tissues. In most cancer types, the expression of ERBB2 was substantially higher in tumour tissues than in normal tissues, which was consistent with the findings of previous analyses in this study (Supplementary Figure 1B).

Furthermore, we investigated the relationship between ERBB2 expression and prognosis in pan-cancer using TCGA data. Cox regression analysis of MRGBP-related survival (OS, DSS, DFI and PFI) indicated that high mRNA expression was a detrimental prognostic factor for ACC (OS: P = 0.038, DFI: P = 0.04), CESC (DFI: P = 0.035; PFI = 0.015), COAD (DFI: P = 0.009), LGG (OS: P < 0.001; DFI: P = 0.003; DSS < 0.001; PFI < 0.001), LUAD (DFI: P = 0.006), OV (OS: P = 0.007; DSS = 0.019), PAAD (OS: P = 0.003; DFI: P = 0.023; DSS =0.038; PFI < 0.001), PCPG (OS: P = 0.024; DSS = 0.001; PFI = 0.002) and UCEC (OS: P < 0.001; DSS = 0.038). However, high mRNA expression was a protective prognostic factor for KICH (OS: P < 0.001; DSS = 0.002; PFI = 0.001), KIRC (OS: P < 0.001; DSS < 0.001; PFI = 0.002), KIRP (DSS = 0.018; PFI = 0.010), MESO (OS: P = 0.003; DSS = 0.024), SARC (PFI = 0.042), STAD (DFI: P = 0.024) and UCS (DSS = 0.04) (Supplementary Figure 1C–1F).

Subsequently, we investigated the association between ERBB2 expression and clinical features in various cancer types. In ACC, TGCT and UCEC, ERBB2 expression was higher in patients with stage III–IV disease than in patients with stage I and II disease. In KIRC and KIRP, ERBB2 expression was higher in patients with stage I–II disease (Supplementary Figure 2). In terms of differences in ERBB2 expression based on tumour grade, ERBB2 expression was found to be higher in patients with G3–G4 LGG and UCEC and lower in patients with G3–G4 CHOL, KIRC and STAD (Supplementary Figure 2). Moreover, patients with recurrent ESCA, PAAD and PCPG had higher ERBB2 expression, whereas those with recurrent BLCA, BRCA, KIRC, KIRP and THYM had lower ERBB2 expression (Supplementary Figure 2).

Furthermore, TIMER2.0 was used to exhibit the landscape of correlation between ERBB2 and various immune infiltrates in human cancers (Supplementary Figure 3). Differences were observed in the regulation of ERBB2 in the immune microenvironment among different tumour types: 1) ERBB2 was positively correlated with B-cell infiltration in ACC, COAD, DLBC, ESCA, HNSC, KICH, PRAD, READ and UCEC but negatively correlated with B-cell infiltration in SKCM, STAD, TGCT, THCA and THYM; 2) ERBB2 was positively correlated with the infiltration of cancer-associated fibroblasts in ACC, BRCA, CESC, DLBC, GBM, LGG, LIHC, LUAD, OV, PCPG, SKCM, TGCT, THYM and UVM but negatively correlated with the infiltration of cancer-associated fibroblasts in ESCA, KIRC and STAD; 3) ERBB2 was positively correlated with the infiltration of resting memory CD4+ T cells in almost all tumour types but negatively correlated with the infiltration of T helper 1 (Th1) cells, Th2 cells and activated memory CD4+ T cells in almost all tumour types; 4) ERBB2 was positively correlated with the infiltration of CD8+ T cells in ACC but negatively correlated with the infiltration of B cells in BRCA, DLBC, SARC, STAD, THCT, THYM and UCEC; 5) ERBB2 was positively correlated with the infiltration of M0 macrophages in LUSC, PAAD, STAD and THCA but negatively correlated with the infiltration of M0 macrophages in KICH, KIRP and KIRC; 6) ERBB2 was positively correlated with the infiltration of M1 macrophages in ACC, LGG and THYM but negatively correlated with the infiltration of M1 macrophages in BLCA, KIRP and KIRC; 7) ERBB2 was positively correlated with the infiltration of M2 macrophages in ACC, BRCA and THYM but negatively correlated with the infiltration of M2 macrophages in KIRP; 8) ERBB2 was positively correlated with the infiltration of neutrophils in almost all tumour types; 9) ERBB2 was positively correlated with the infiltration of NK cells in ACC, SKCM and THYM but negatively correlated with the infiltration of NK cells in CESC, ESCA and GBM; 10) ERBB2 was negatively correlated with the infiltration of γδT cells in almost all tumour types; 11) ERBB2 was positively correlated with the infiltration of regulatory T cells (Tregs) in CESC, COAD, ECSA, HNSC, LIHC, LUAD and LUSC but negatively correlated with the infiltration of Tregs in KIRC.

Similarly, ERBB2 expression was negatively correlated with many typical immune pathways in multiple human cancers (Figure 2A). Moreover, the correlation between ERBB2 expression and metabolic pathways was different in various tumours. ERBB2 expression was significantly positively correlated with metabolic pathways in BLCA, BRCA, ESCA, HNSC, LIHC, PAAD, PRAD and STAD but negatively correlated with metabolic pathways in CHOL, KIRP and THCA (Figure 2B).
