# Supplementary figures and images for: Integration of transcriptomics, proteomics, and metabolomics data to reveal HER2-associated metabolic heterogeneity in gastric cancer with response to immunotherapy and neoadjuvant chemotherapy
Source: Front Immunol. 2022 Aug 4;13:951137. doi: 10.3389/fimmu.2022.951137 (PMC9389544; doi:10.3389/fimmu.2022.951137)

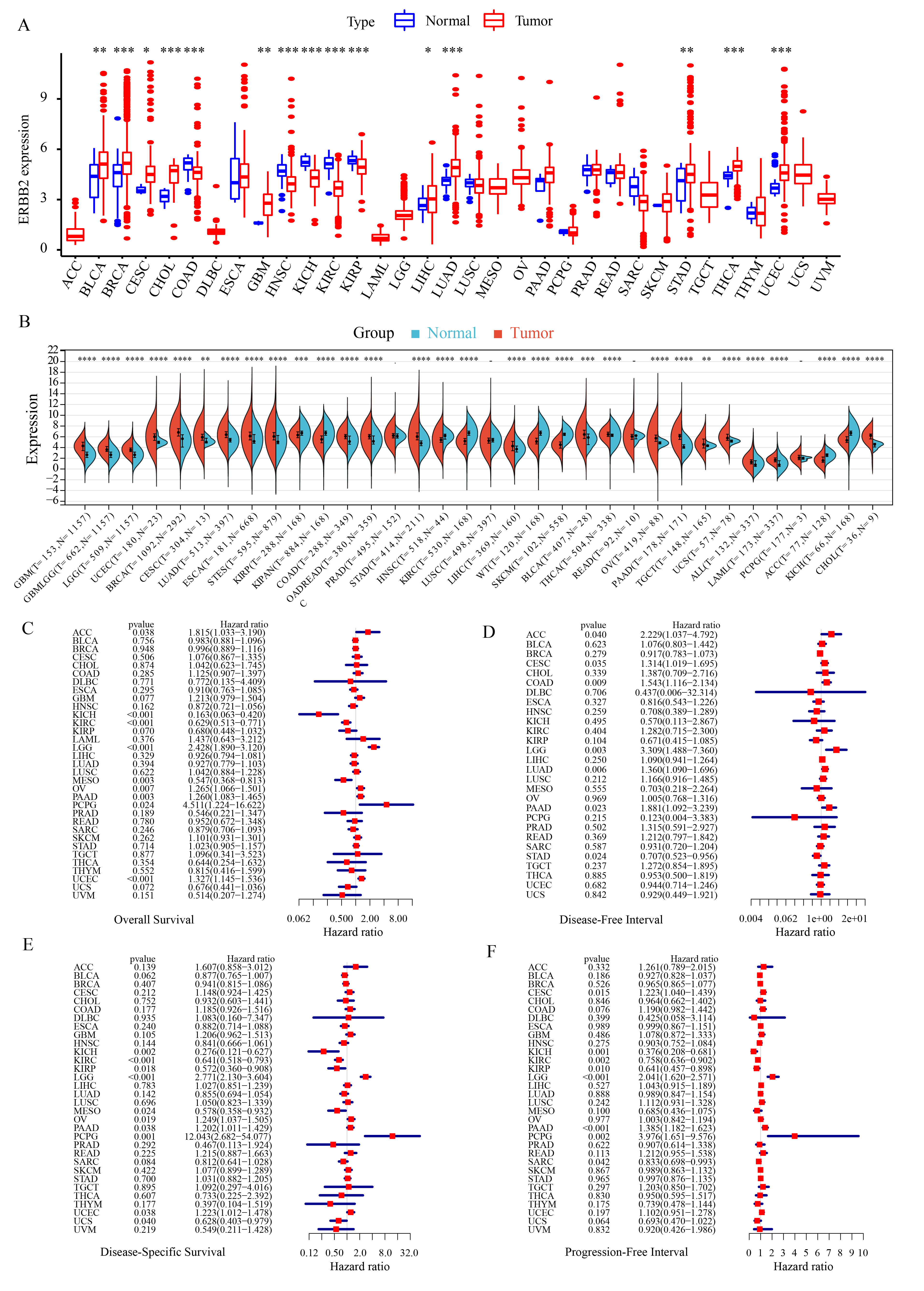

Supplement: Supplementary Figure 1 — The mRNA expression profiles and prognostic performance of ERBB2 in pan-cancer. (A) The mRNA expression of ERBB2 between tumour and healthy tissues was assessed using tissues from TCGA database. (B) Comparison of ERBB2 expression levels between tumour tissues from TCGA database and healthy tissues from the GTEx database. Relationship between ERBB2 expression and overall survival (C), disease-free interval (D), disease-specific survival (E) and progression-free interval (F) in pan-cancer. [file Image_1.jpeg]

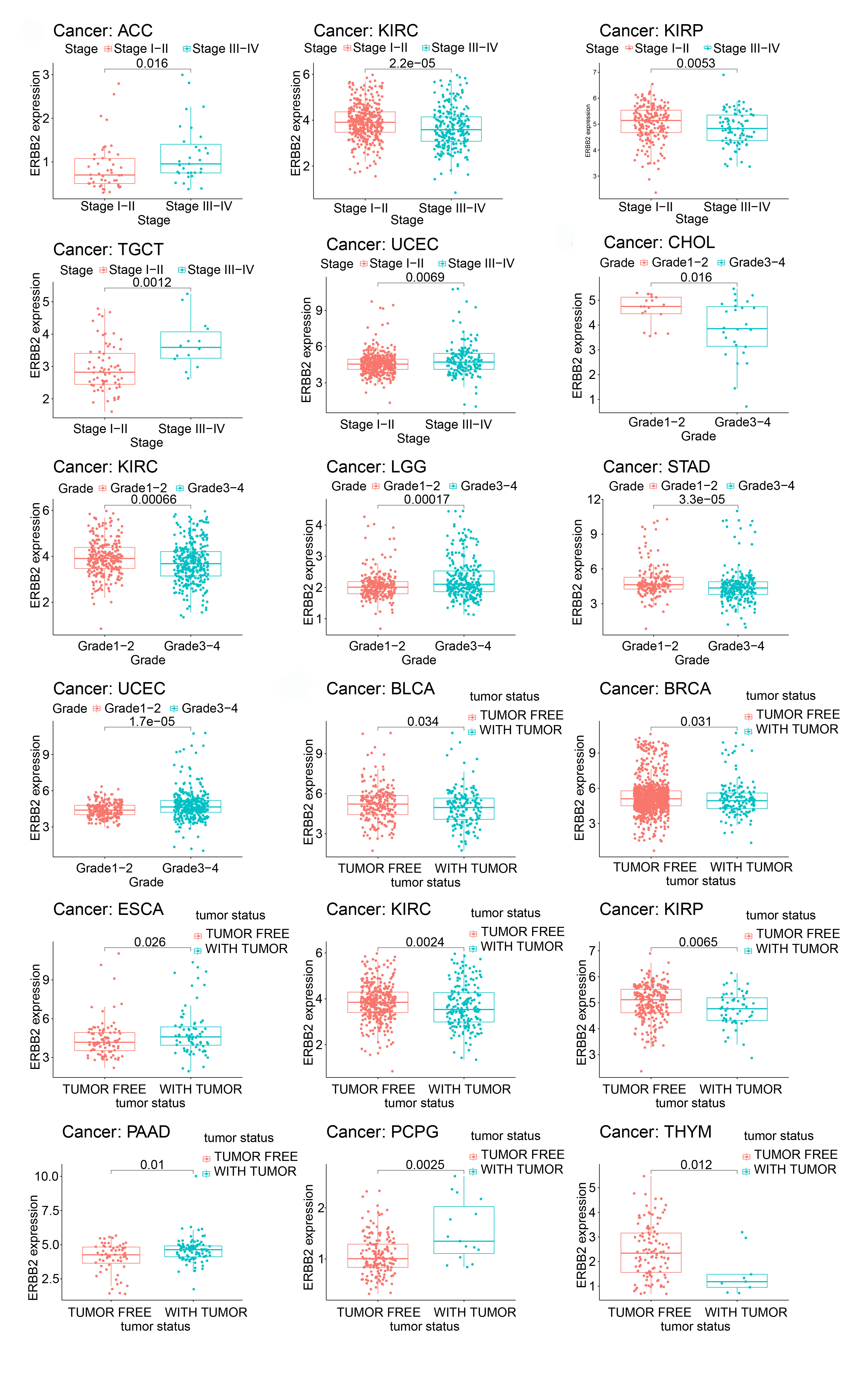

Supplement: Supplementary Figure 2 — Correlation between ERBB2 expression and tumour stage, tumour grade and tumour status. [file Image_2.jpeg]

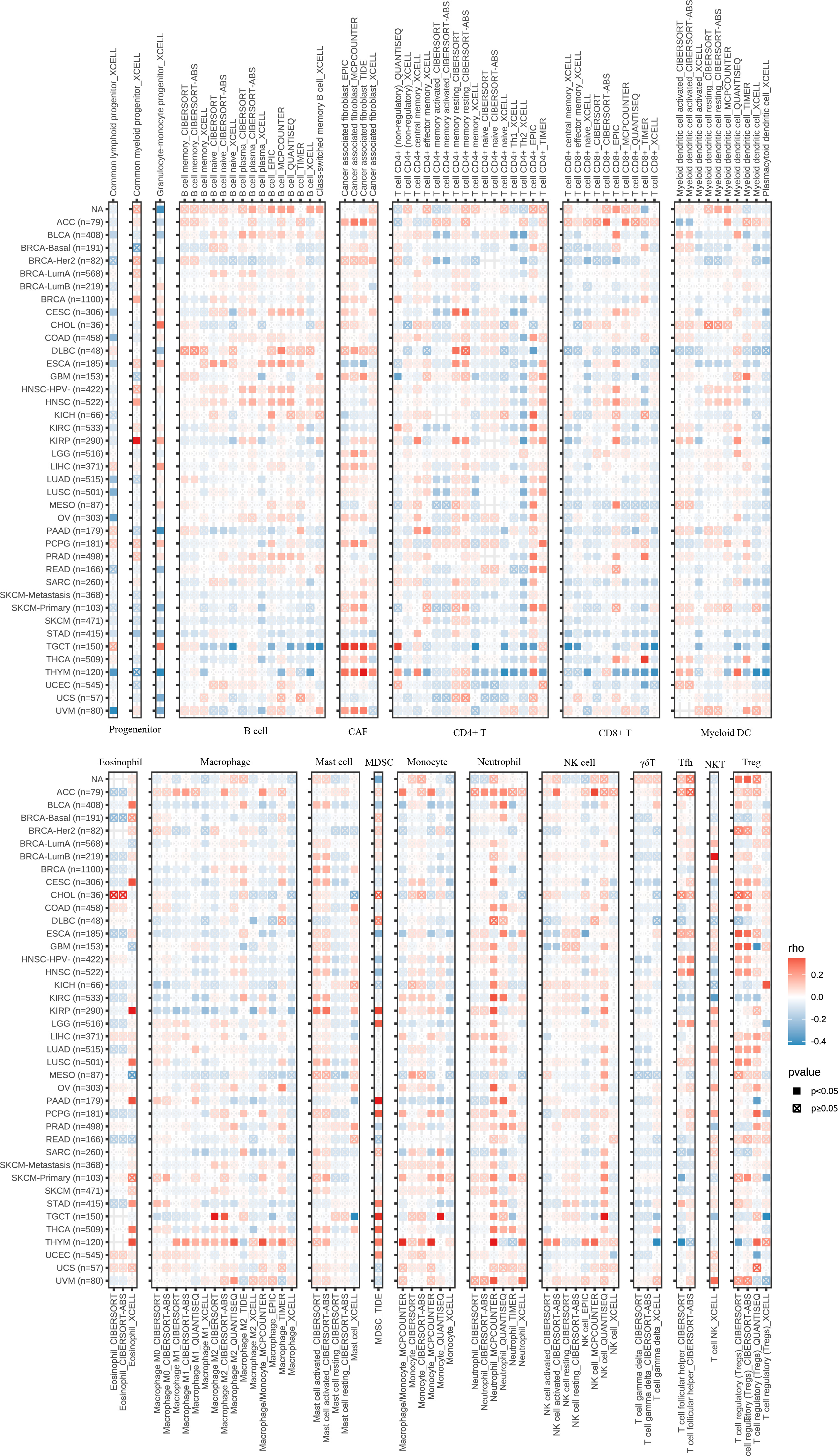

Supplement: Supplementary Figure 3 — Correlation between ERBB2 expression and immune infiltration in pan-cancer. [file Image_3.jpeg]

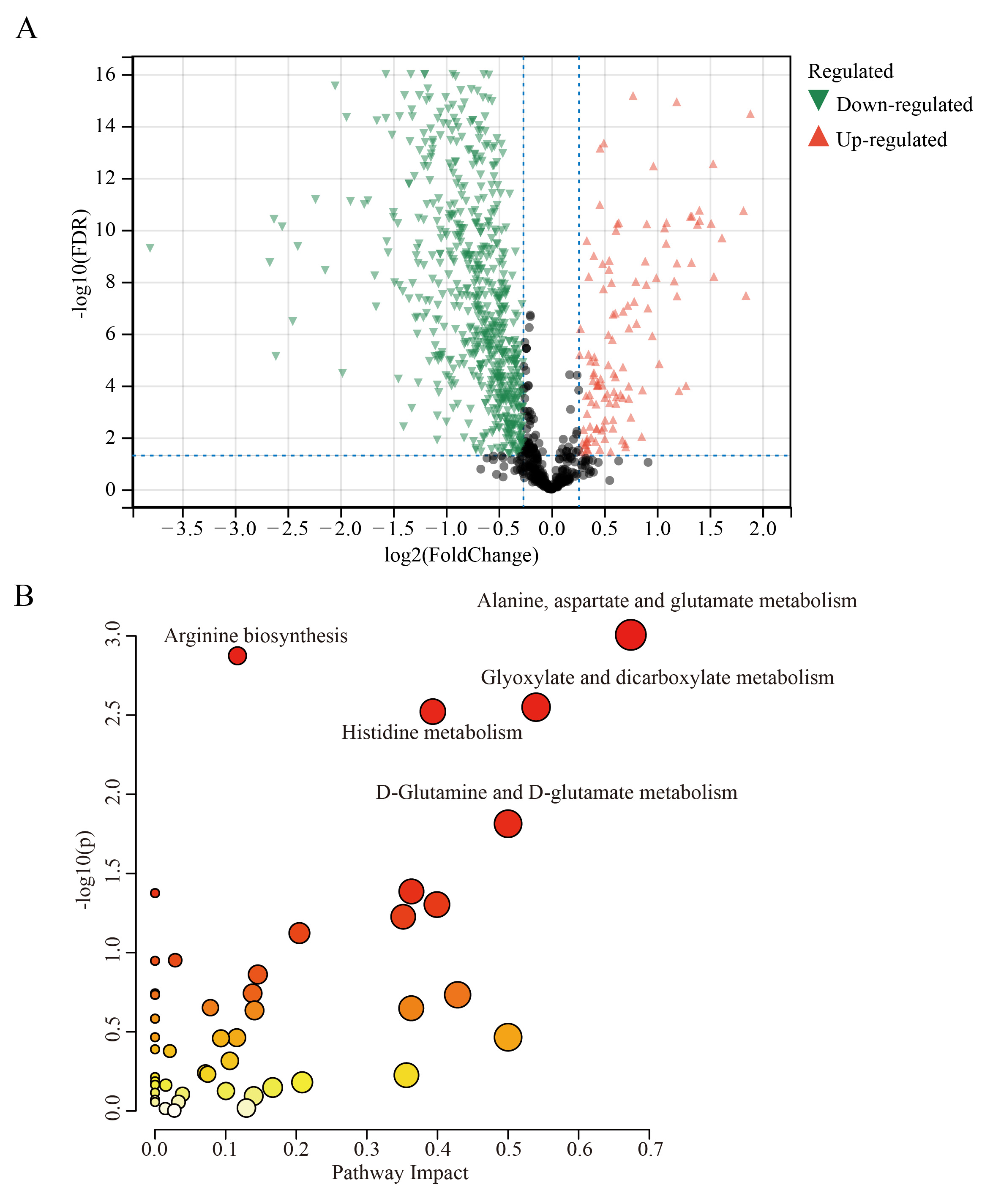

Supplement: Supplementary Figure 4 — Identification of differentially expressed metabolites and their related metabolic pathways. (A) Volcano plot demonstrating the expression profile of 1165 metabolites; a total of 859 metabolites exhibited significant differential abundance (FDR < 0.05, fold change > 1.2 or < 5/6) in the peripheral blood samples of patients with GC and healthy volunteers. (B) Topology analysis of dysregulated metabolic pathways associated with the occurrence of GC. The size of the bubble area denotes the impact of each pathway, with a colour representing significance from the highest in red to the lowest in white. [file Image_4.jpeg]

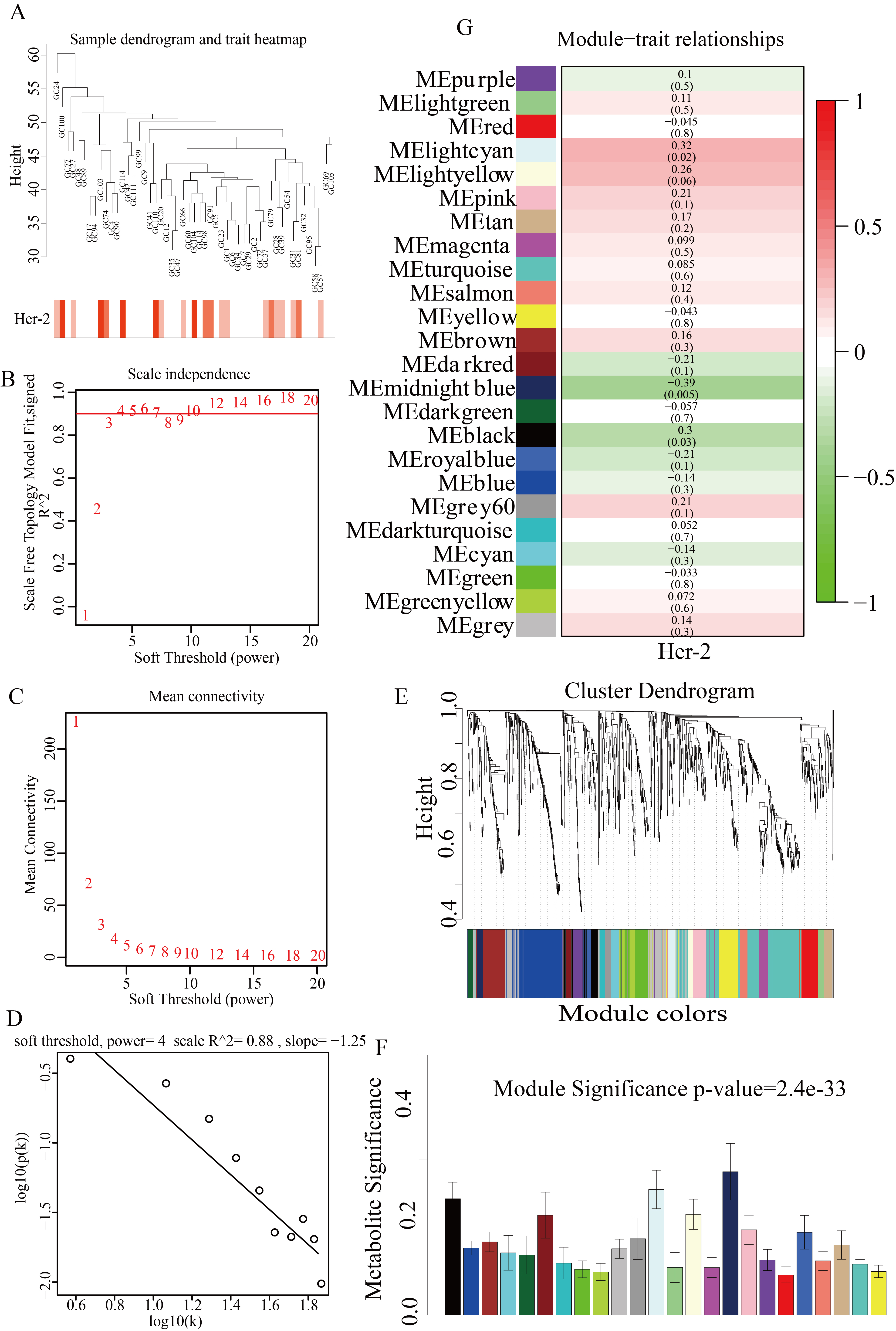

Supplement: Supplementary Figure 5 — Detailed processes of weighted metabolite co-expression network analysis. (A) Clustering dendrogram of 51 samples to detect outliers (white-to-red linear gradient colour associated with corresponding HER2 expression). (B) The scale-free fit index for soft-thresholding powers (β). (C) Mean connectivity calculation. (D) Evaluation of scale-free topology with a β of 4. (E) Hierarchical clustering dendrograms of the identified coexpressed metabolites in specific modules of GC. (F) Distribution of average metabolite significance and errors in modules associated with HER2 expression. (G) Heatmap demonstrating the correlation between module eigenmetabolites and HER2 expression. [file Image_5.jpeg]

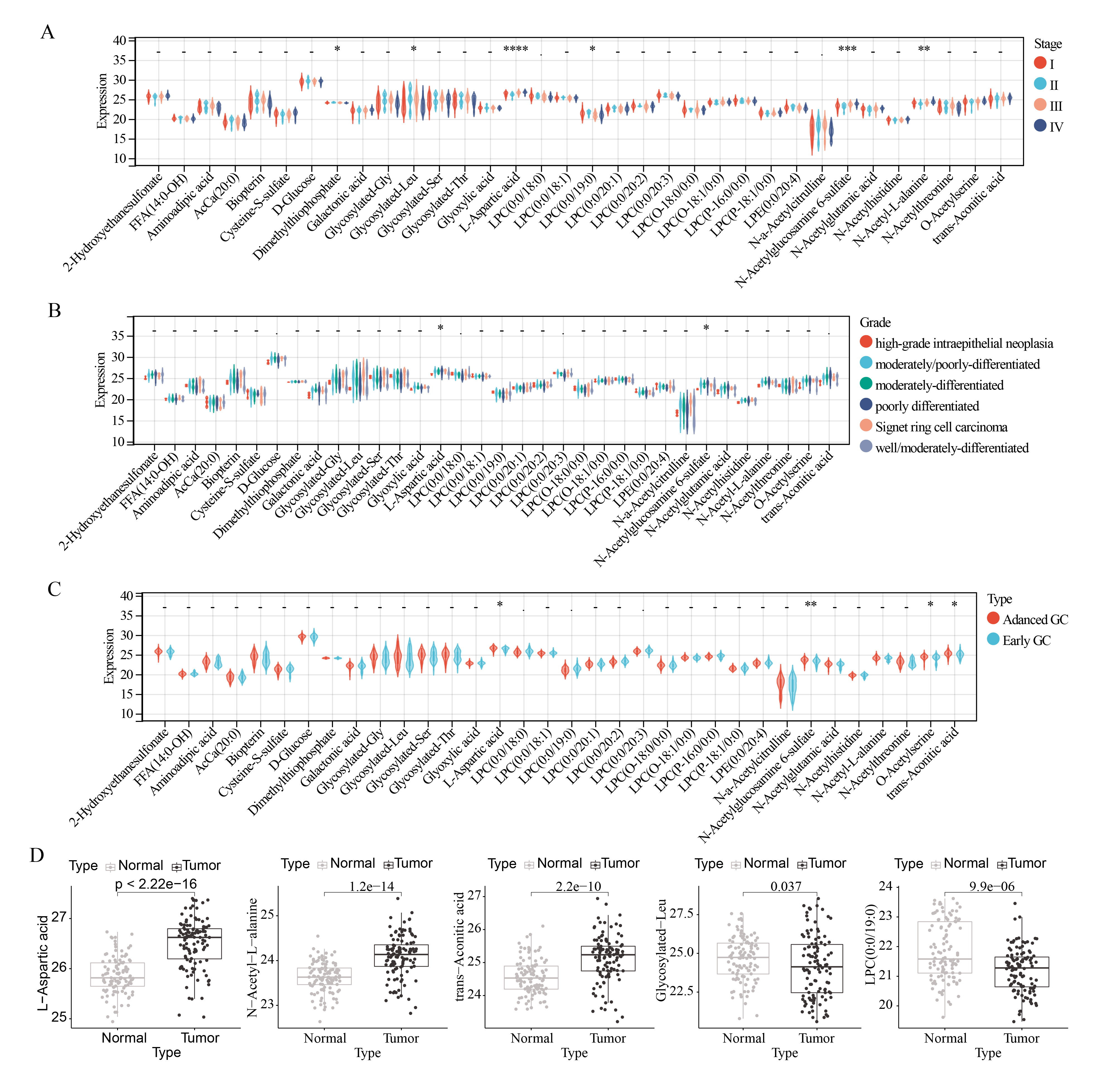

Supplement: Supplementary Figure 6 — Identification of differentially expressed hub metabolites significantly associated with GC stage, grade and type. Correlation between 34 HER2-coexpressed metabolites and tumour (A) stage, (B) grade and (C) type. (D) The expression distribution of the HER2-coexpressed metabolites associated with tumour stage, grade and type in tumour and healthy samples. Only metabolites with a p-value of <0.05 are demonstrated. [file Image_6.jpeg]

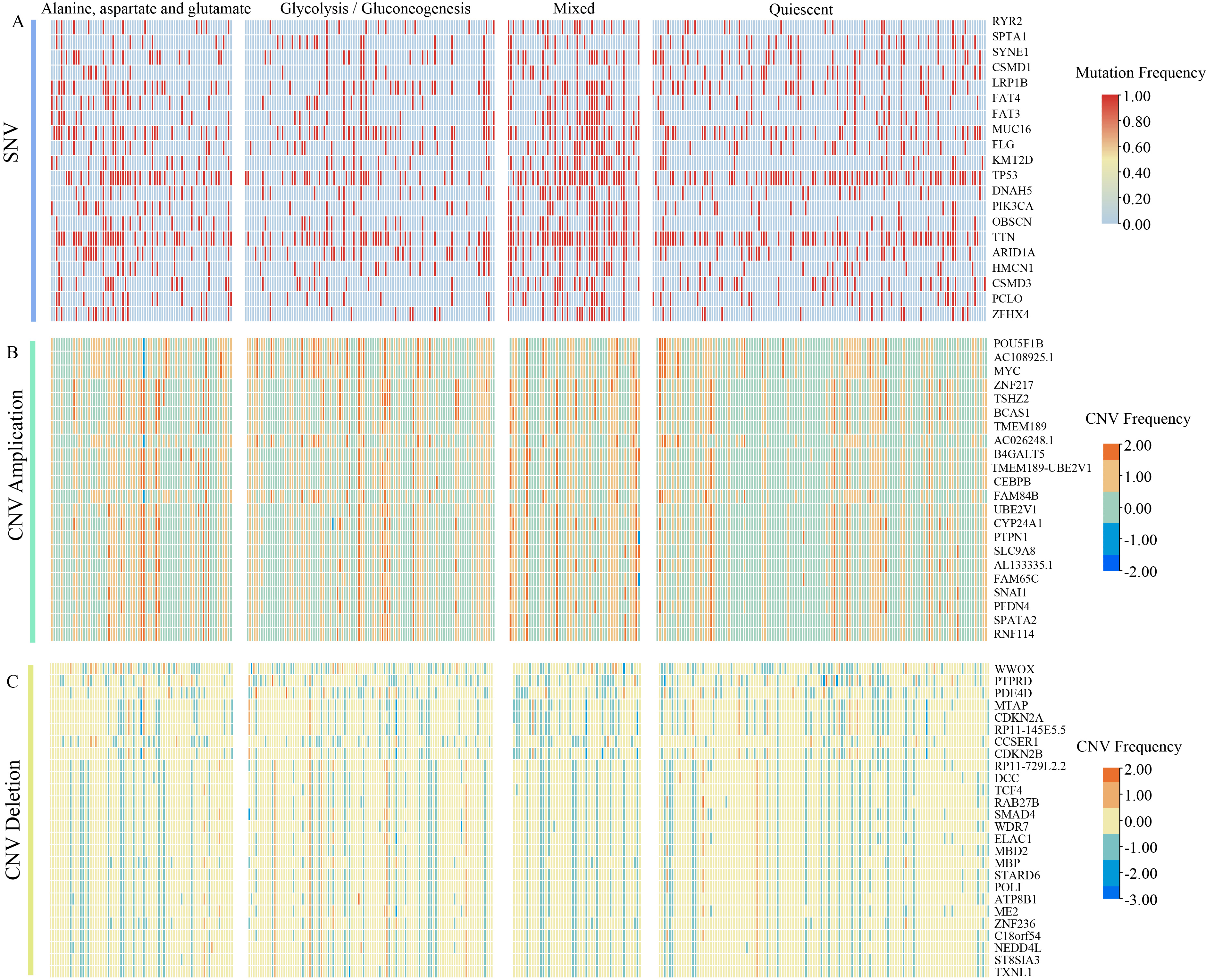

Supplement: Supplementary Figure 7 — Panoramic view of the (A) SNV, (B) CNV amplification and (C) CNV deletion frequencies of the top 20 mutated genes across the four metabolic subtypes. [file Image_7.jpeg]

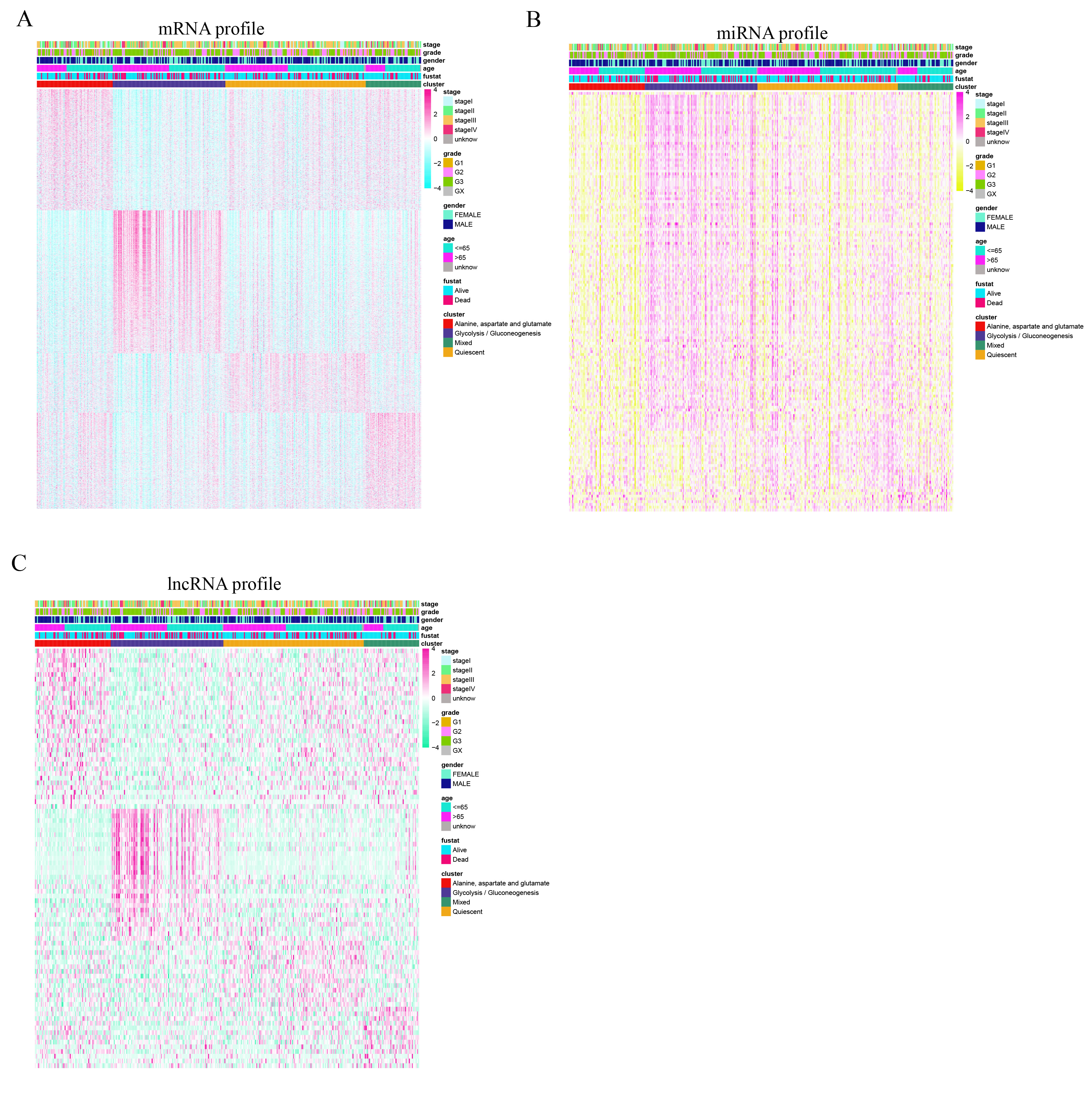

Supplement: Supplementary Figure 8 — Panoramic view of the specific molecules (A: mRNA, B: miRNA, C: lncRNA) of each metabolic subtype. [file Image_8.jpeg]

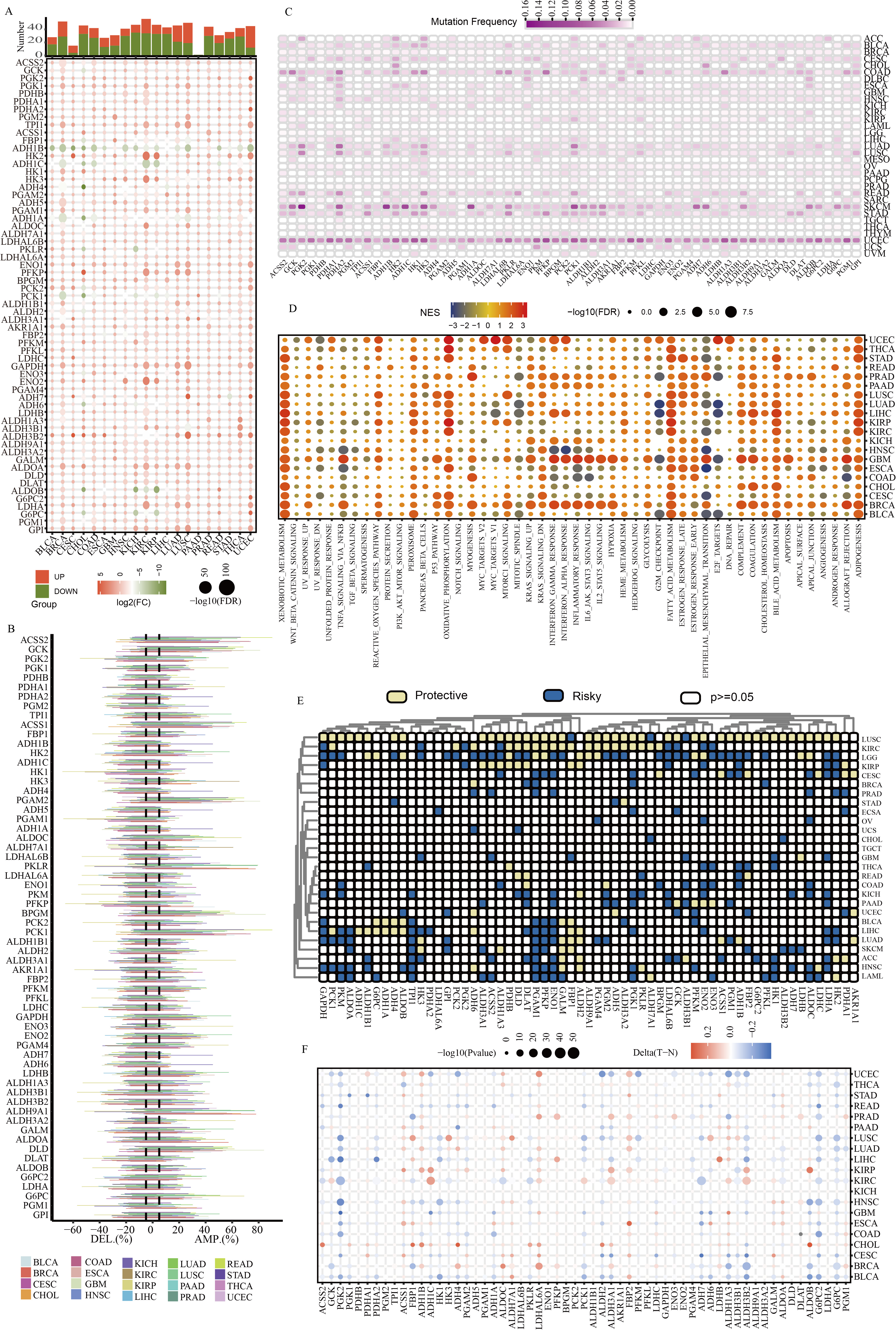

Supplement: Supplementary Figure 9 — Panoramic view of glycolysis/gluconeogenesis metabolism-related genes in pan-cancer. (A) Changes in the mRNA expression of glycolysis/gluconeogenesis metabolism-related genes across cancer types. The frequencies of copy number variation (B) and single-nucleotide variation (C) in diverse types of cancers. (D) Enrichment analysis for cancer pathway signalling between tumour samples with high and low enrichment scores of the genes. (E) Survival landscape of the genes across cancer types. (F) Heatmap demonstrating the methylation levels of the genes across cancer types. [file Image_9.jpeg]

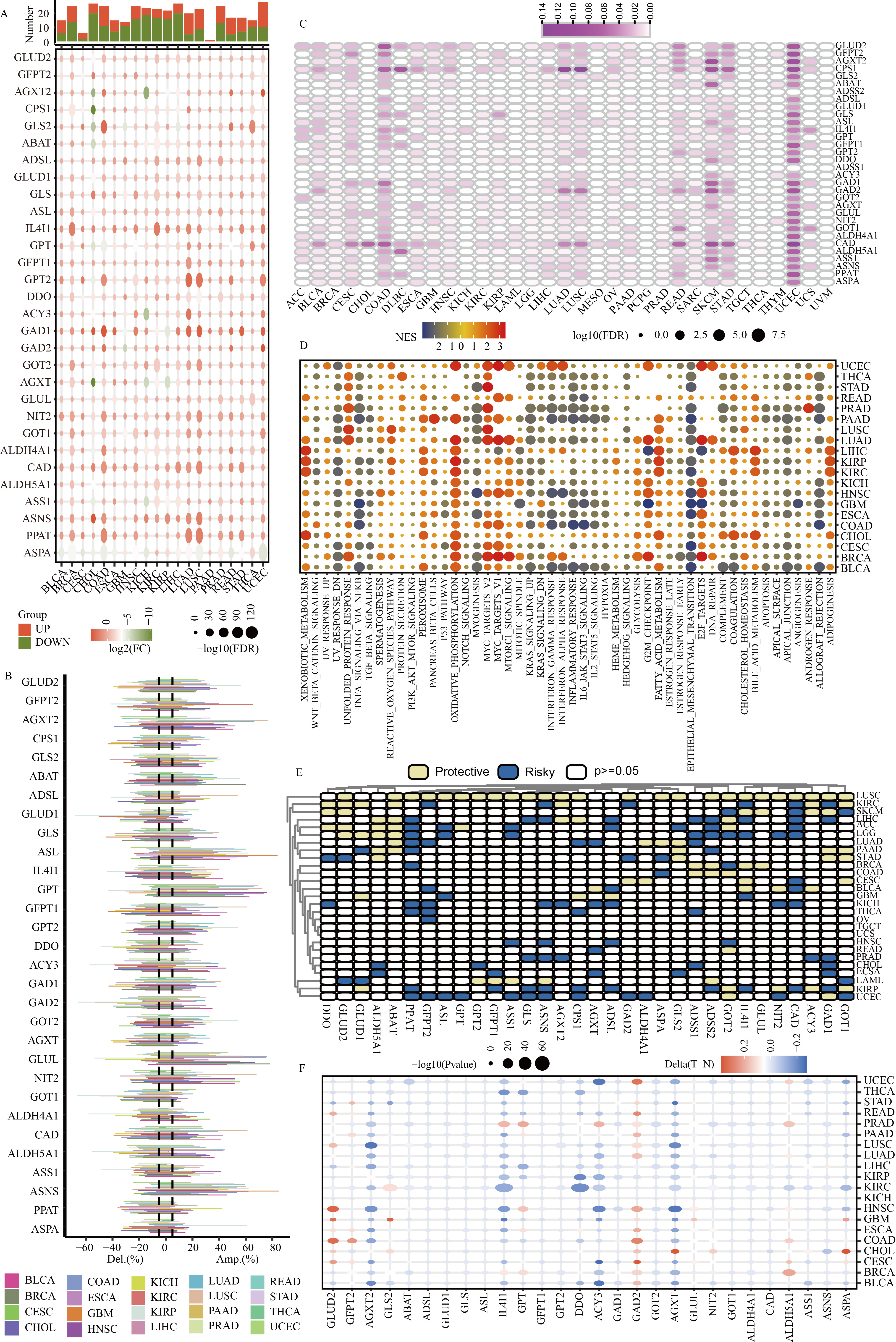

Supplement: Supplementary Figure 10 — Panoramic view of alanine–aspartate–glutamate metabolism-related genes in pan-cancer. (A) Changes in the mRNA expression of alanine–aspartate–glutamate metabolism-related genes across cancer types. The frequencies of copy number variation (B) and single-nucleotide variation (C) in diverse types of cancers. (D) Enrichment analysis for cancer pathway signalling between tumour samples with high and low scores of the genes. (E) Survival landscape of the genes across cancer types. (F) Heatmap demonstrating the methylation levels of the genes across cancer types. [file Image_10.jpeg]

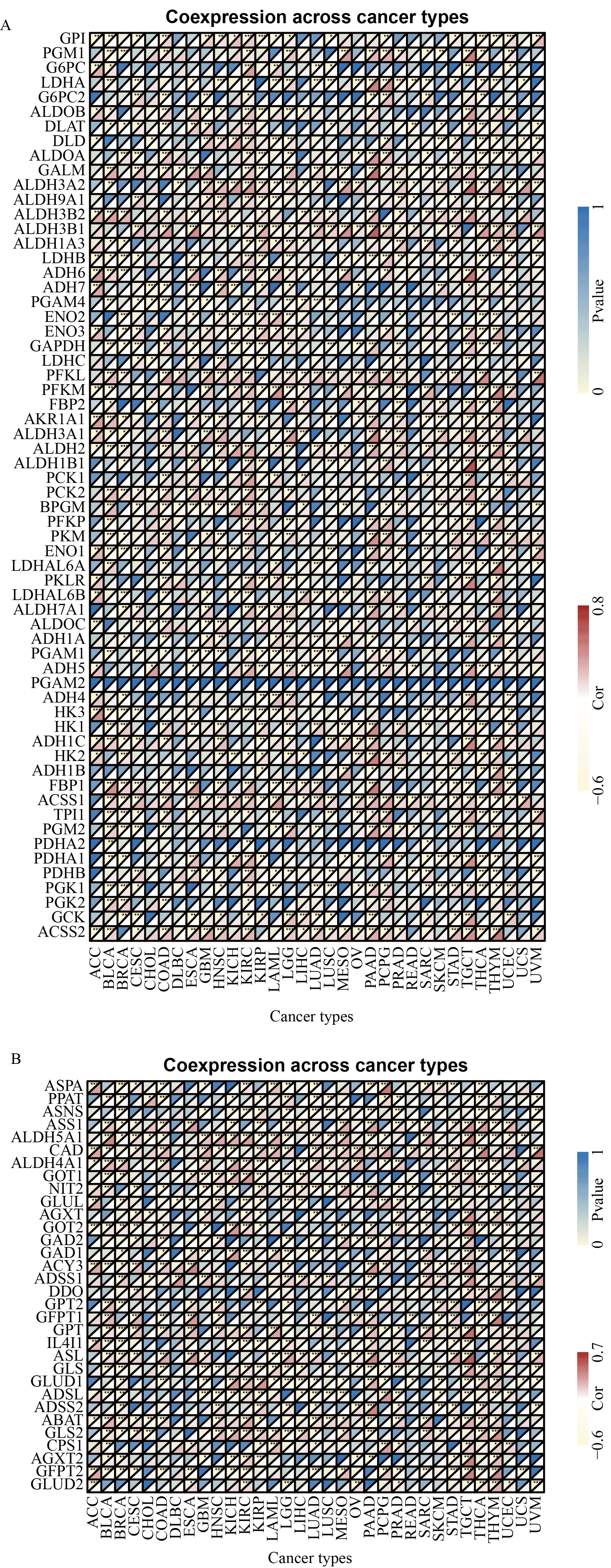

Supplement: Supplementary Figure 11 — Relationship between ERBB2 expression and glycolysis/gluconeogenesis (A) and alanine–aspartate–glutamate (B) metabolism. [file Image_11.jpeg]
